# Transparency about the outcomes of mental health services (IAPT approach): an analysis of public data

**DOI:** 10.1016/S0140-6736(17)32133-5

**Published:** 2018-02-17

**Authors:** David M Clark, Lauren Canvin, John Green, Richard Layard, Stephen Pilling, Magdalena Janecka

**Affiliations:** a Department of Experimental Psychology, University of Oxford, Oxford, UK; b The Oxford Academic Health Sciences Network, Oxford, UK; c Department of Clinical Health Psychology, Central and North West London National Health Service Trust, London, UK; d Centre for Economic Performance, London School of Economics, London, UK; e Department of Clinical, Educational and Health Psychology, University College London, London, UK; f Seaver Autism Center, Department of Psychiatry, Icahn School of Medicine at Mount Sinai, New York, NY, USA

## Abstract

**Background:**

Internationally, the clinical outcomes of routine mental health services are rarely recorded or reported; however, an exception is the English Improving Access to Psychological Therapies (IAPT) service, which delivers psychological therapies recommended by the National Institute for Health and Care Excellence for depression and anxiety disorders to more than 537 000 patients in the UK each year. A session-by-session outcome monitoring system ensures that IAPT obtains symptom scores before and after treatment for 98% of patients. Service outcomes can then be reported, along with contextual information, on public websites.

**Methods:**

We used publicly available data to identify predictors of variability in clinical performance. Using β regression models, we analysed the outcome data released by National Health Service Digital and Public Health England for the 2014–15 financial year (April 1, 2014, to March 31, 2015) and developed a predictive model of reliable improvement and reliable recovery. We then tested whether these predictors were also associated with changes in service outcome between 2014–15 and 2015–16.

**Findings:**

Five service organisation features predicted clinical outcomes in 2014–15. Percentage of cases with a problem descriptor, number of treatment sessions, and percentage of referrals treated were positively associated with outcome. The time waited to start treatment and percentage of appointments missed were negatively associated with outcome. Additive odd ratios suggest that moving from the lowest to highest level on an organisational factor could improve service outcomes by 11–42%, dependent on the factor. Consistent with a causal model, most organisational factors also predicted between-year changes in outcome, together accounting for 33% of variance in reliable improvement and 22% for reliable recovery. Social deprivation was negatively associated with some outcomes, but the effect was partly mitigated by the organisational factors.

**Interpretation:**

Traditionally, efforts to improve mental health outcomes have largely focused on the development of new and more effective treatments. Our analyses show that the way psychological therapy services are implemented could be similarly important. Mental health services elsewhere in the UK and in other countries might benefit from adopting IAPT's approach to recording and publicly reporting clinical outcomes.

**Funding:**

Wellcome Trust.

## Introduction

In most countries, if you have a mental health problem patients cannot obtain information about the clinical outcomes achieved by the psychological therapy service that they might be considering for treatment. Additionally, when a service does hold such data, they will not usually be made public. This absence of transparency is a disservice to patients. It is also an impediment to the development of more effective health care, because it makes it difficult to study, and learn from, the variation in service outcomes.

The Improving Access to Psychological Therapies (IAPT) programme^1^ is a rare exception to the general absence of transparency about outcomes for mental health services. Starting in 2008, the UK Government developed a plan to expand access to evidence-based psychological therapies for depression and anxiety disorders by training a new workforce and deploying it in specialised services throughout England. Every local health area (otherwise known as a clinical commissioning group; CCG) now has an IAPT service, which provides psychological treatments in line with the stepped-care clinical guidelines^2^, ^3^ issued by the UK's National Institute for Health and Care Excellence (NICE). For all anxiety disorders, cognitive behavioural therapy (CBT) is recommended. For depression, a wider range of treatments (CBT, counselling, couples therapy, interpersonal therapy, and brief psychodynamic therapy) are recommended. The latest data^2^ show that around 950 000 people a year have an initial assessment and advice from the IAPT service, with more than 537 000 going on to have a course of therapy (defined as two or more sessions), with the predominant method being CBT.^4^, ^5^ A distinctive feature of IAPT is the use of an outcome monitoring system^6^ that ensures 98% of patients have scores recorded on well-validated self-report measures of depression and anxiety at the beginning and end of treatment, with CCG level summaries of such data publicly available. This system is a great improvement on the national situation before the start of IAPT. At that time, a survey^7^ found that only 38% of patients had an assessment of their symptoms at the beginning and end of treatment.

**Research in context**

**Evidence before this study**

Many randomised controlled trials have shown that psychological therapies are effective interventions for a wide range of mental health conditions. On the basis of these trials, the National Institute of Health and Care Excellence (NICE) now recommends certain psychological therapies as first-line interventions for common mental health conditions such as depression and the anxiety disorders.

However, a large gap exists between recommendation and implementation. In particular, in most countries routine psychological therapy services do not record and publish their clinical outcomes, which makes it difficult to know if outcomes are in line with expectation and hinders attempts to study, and learn from, between-service variation in clinical outcomes.

The English Improving Access to Psychological Therapies (IAPT) programme is an exception. IAPT services treat more than 537 000 patients with depression or anxiety disorders each year using NICE-recommended psychological therapies. A unique outcome monitoring system allows IAPT to gather outcome data for 98% of treated patients. Since May 2012, the clinical performance of each service has been reported on public websites. We searched MEDLINE with search terms “IAPT” and “outcome” for articles published from May, 2012, to November, 2017. Only one article, which focused on social deprivation, had used the national public data to predict outcomes. None had investigated service organisation.**Added value of this study** We describe the IAPT outcome monitoring system, discuss why it is so effective, and illustrate the value of the publicly available data by construction of statistical models to predict local variation in outcomes in 2014–15. We also tested the robustness of the models by using them to predict changes in outcomes from one year to the next. We identified five aspects of the organisation of a service that are associated with improved clinical outcomes.

**Implications of all the available evidence**

Traditionally, clinicians have been sceptical about the possibility of obtaining outcome data for most people who are treated in routine psychotherapy services. We show that getting such data is possible and describe a system that could be applied in other countries. To date, efforts to improve the outcomes achieved with psychotherapies have mainly focused on the development of new and more effective treatments. Our analyses suggest that the way in which psychological therapy services are implemented could be similarly important. The findings open up new possibilities for the improvement of mental health care.

To monitor IAPT outcomes, patients complete brief measures of depression and anxiety every session so that a symptom score for after treatment is available even if patients complete therapy earlier than expected. Therapists make use of the measures in treatment planning and supervision. IAPT services have specialised information technology systems that record patient data and make it available to therapists, supervisors, and managers. This session-by-session approach to outcome monitoring was successfully piloted in a community therapy service for victims of the Omagh bomb^8^ (County Tyrone, Northern Ireland) and was enhanced for use in IAPT.^9^

IAPT gathers detailed information about patients, their course of treatment, and clinical outcomes.^9^ Once a month these data are sent to National Health Service (NHS) Digital, which issues regular reports for the number of people accessing services and their outcomes, along with a range of process variables (eg, average number of sessions). The most complete dataset appears in the annual reports. Most data provided by NHS Digital are also available in Public Health England's Common Mental Health Disorders Profiling Tool, along with other contextual information about CCGs (eg, social deprivation score).

In this Article, we aimed to illustrate the value of information from IAPT's outcome monitoring system by using public data from the websites to identify organisational and other characteristics of services with better and worse outcomes.

## Methods

### IAPT outcome monitoring system

IAPT reports clinical outcomes for all patients who have had at least two sessions of treatment and have been discharged. Around 81% of people who are believed suitable for treatment have two or more sessions^4^ (appendix). We used publicly available data to identify the characteristics of IAPT services that achieve better and worse clinical outcomes. We analysed the outcome data released by NHS Digital and Public Health England for the 2014–15 financial year (April 1, 2014, to March 31, 2015) and developed a predictive model. We then waited until the IAPT data for 2015–16 (April 1, 2015, to March 31, 2016) were released and tested whether the identified predictors replicated in the new dataset. We also tested whether change in predictors identified in the 2014–15 model was associated with change in outcomes between 2014–15 and 2015–16. Similarity of findings in analyses of between-service variation at a particular time and within service change over time would strengthen the argument that the identified predictors have a causal role because spurious third variables are unlikely to be similar in the two types of analysis.

### Measures of clinical outcome

The Patient Health Questionnaire 9-item (PHQ-9) score^10^ (clinical cutoff >9) is used to measure symptoms of depression. The Generalised Anxiety Disorder 7-item (GAD-7) score^11^ (cutoff >7) is the default measure of anxiety but services can also use more specific measures for particular anxiety disorders.^9^ We modelled two of the standard outcome indices included in NHS Digital's reports: the proportion of patients in a CCG who have reliably improved and the proportion who have reliably recovered. Patients are reliably improved if their scores on depression or anxiety, or both, have reduced by a reliable amount^9^, ^12^ (ie, more than the measurement error of the scale) and neither measure has shown a reliable increase. Patients are reliably recovered if they reliably improve and their scores on both depression and anxiety are below the clinical cutoff scores^9^ at the end of treatment. We assumed that patients (2%) without scores after treatment had not improved or recovered. The total treated cohort is the denominator for calculation of reliable improvement. Only cases above the clinical cutoffs^9^ for depression, anxiety, or both before treatment are included in the computation of reliable recovery. The scores used to calculate reliable improvement and recovery are the last available after baseline scores, usually from the final therapy session but occasionally from an earlier session. NHS Digital does not report how often earlier session scores are used but data from an NHS Trust that provides multiple IAPT services suggest that the use of such scores is rare (2·5%; Green J, CNWL NHS Trust, personal communication).

### Possible predictors of outcome

Six possible predictors were investigated. The first predictor was the percentage of treated patients for whom a problem descriptor (ICD-10 code)^13^ was recorded. This variable was regarded as important because the type of treatment a patient should receive is based on ICD-10 codes.^13^ The second was the percentage of referrals who were treated, because this variable captures the extent to which services focus on treatment, as opposed to providing only assessment, advice, and signposting. The third was the percentage of missed appointments (patient did not attend and failed to give advance warning). The fourth was the number of days patients waited between referral and starting treatment, the fifth was the number of treatment sessions, and the sixth was the Index of Multiple Deprivation (IMD),^14^ for the area. The IMD covers seven domains, including income, employment, barriers to services, and crime. We did not use a seventh possible predictor (number of NICE-recommended depression treatments that the service could deliver) in the main analyses because preliminary analysis showed the NICE predictor did not relate to outcome. For the third and fifth predictors, in 2014–15, data were available only for the last 3 months of the study. For other predictors, data covering the full periods were used. The IMD values are from September, 2015, and represent the latest available figures.

### Statistical analyses

Analyses of predictors of IAPT performance per CCG during single years were done in *R* software (version 3.2.2) with β regression^15^ (betareg package),^16^ as recommended for models with continuous but bounded outcomes (eg, proportions, with all data points falling between 0 and 1).^16^ The method exploits the flexibility of β distribution, which accommodates data heteroscedasticity (unequal variance) and absence of symmetry around the mean, both common features of proportions data that are less well dealt with in commonly used logistic and linear regression models. A logit link function in β regression renders the coefficients interpretable in terms of odds ratios (ORs). Analyses were initially run on the 2014–15 data and then repeated on the 2015–16 data.

To explore the associations between predictor and outcome variables, we first fitted simple regressions. Then we fitted a multiple regression model to each of the outcomes, entering all predictors that were independently significantly associated with the outcome, which allowed us to assess the joint contribution of the predictors and account for possible correlations between them. We examined goodness of fit for multiple regression models using half normal plots of deviance residuals (plots of absolute values of standardised residuals). To detect any outliers that could bias our results, we identified CCGs with an especially large effect over model fit using Cook's distance statistics. We inspected the characteristics of these CCGs, and residual plots before and after the exclusions, to decide whether to retain these CCGs in the final models (appendix). Up to two CCGs (<1% of the total sample) were excluded from a model when justified by these indices. We then plotted the results, and curves were fitted with the LOESS (locally weighted smoothing) function. Finally, for each predictor, we estimated the increase in the odds of improvement or recovery if the worst-performing CCG matched the best-performing CCG on that predictor.

We used ranges of predictor values recorded across the CCGs, and the ORs derived from the β regressions. Because ORs are additive on the log scale, the formula for these calculations was exp (log[OR] × range). For example, if the difference between worst-performing and best-performing CCG on a predictor was 100 units, and a unit of that predictor was associated with the odds of recovery of 1·001, the formula would be exp (log[1·001] × 100), producing an additive OR of 1·105. This OR value suggests that patients in the worst-performing CCG would be 10·5% more likely to recover if this CCG were to match the highest values of this predictor recorded in the sample. To facilitate interpretation for all predictors that were negatively associated with outcome, we present the reciprocal of the original ORs (ie, original OR of 0·75 is presented as OR 1·33), which is in line with published recommendations^17^ to report ORs standardised to less than 1. For those variables, the possible gains in outcome indicated by the additive ORs would result from a decrease in the predictor value.

We also investigated whether changes in predictors between 2014–15 and 2015–16 were related to changes in clinical outcomes between the 2 years. For each CCG, we computed Δ scores, which relate to differences between a variable's value in 2014–15 and in 2015–16. We first compared the mean Δ scores of our predictors in the 10% of CCGs that improved most, the 10% that improved least or deteriorated, and the overall mean. To formally test these findings, we ran linear regressions using the Δ scores of predictors and outcomes, because outcomes were no longer proportion scores and were normally distributed. To identify the strongest predictors and obtain standardised indices of the variance explained by correlated predictors, we computed semi-partial correlations in full models (package ppcor), which indicate correlations between the outcome and any given predictor, while controlling for the effects of all other predictors. Although IMD did not change, we included absolute IMD score in all models to account for the possibility that social deprivation might affect the degree of change in clinical performance.

### Validation procedures

To mitigate against any bias due to excessive statistical power or other data artifacts, we repeated all analyses in permuted datasets—ie, when the associations between outcomes and predictors were disrupted by random sampling. To further investigate whether associations identified with our preferred method of β regression were robust, we repeated the analyses using standard logistic regression. Both validation analyses suggest our results arise from true associations between outcomes and predictors, rather than any analytical artifacts (appendix).

### Role of the funding source

The funder of the study had no role in study design, data collection, data analysis, data interpretation, or writing of the report. The corresponding author had full access to all the data in the study and had final responsibility for the decision to submit for publication.

## Results

In 2014–15, 211 CCG-based IAPT services (table 1) treated 468 881 patients.^5^ In 2015–16, mergers of some neighbouring CCGs resulted in 209 CCG-based IAPT services (table 1), which together treated 537 131 patients.^4^

**Table 1:** Descriptive statistics for the CCG sample in 2014–15 and 2015–16

	**2014–15**			**2015–16**		
	CCG (n)	Mean (SD)	Median (range)	CCG (n)	Mean (SD)	Median (range)
Reliable improvement (%)	211	60·60% (7·80)	61·50% (24·80–76·80)	209	62·47% (6·63)	62·50% (35·40–80·10)
Reliable recovery (%)	211	42·87% (7·50)	43·00% (17·60–64·60)	209	44·44% (6·34)	44·60% (20·40–58·70)
Patients finishing treatment (n)	211	2217 (1424)	1830 (335–10 470)	209	2567 (1579)	2220 (510–10 430)
Patients finishing treatment classified as a clinical case at pretreatment (n)	211	1978 (1289)	1615 (270–9650)	209	2330 (1436)	2020 (465–9635)
Patients with problem descriptor completeness (%)	211	67·75% (29·87)	75·80% (0–100)	209	76·17% (25·66)	85·07% (0–100)
Patients who enter treatment and receive a course of treatment (%)	211	58·28% (12·94)	58·32% (19·44–90·84)	209	57·74% (12·87)	57·35% (26·54–88·29)
Missed appointments (%)	208	11·30% (4·10)	10·36% (4·48–27·27)	209	11·87% (4·17)	10·87% (3·65–25·62)
Treatment appointments (n)	208	6·35 (0·98)	6·33 (4·23–8·83)	209	6·41 (0·91)	6·34 (3·99–8·62)
Days before entering treatment	211	33·73 (20·56)	28·10 (6·70–124·10)	209	30·98 (23·26)	23·60 (5·90–139·30)
Index of Multiple Deprivation	208	21·96 (8·71)	20·65 (5·80–47·40)	208	21·96 (8·71)	20·65 (5·80–47·40)

CCG=clinical commissioning group.

In 2014–15, all potential predictors were significantly associated with reliable improvement and recovery when considered on their own (table 2), and for reliable recovery, all predictors were significant in multiple regression (table 2). Percentage of cases with a problem descriptor, the mean number of treatment sessions, and the percentage of referred patients who received a course of treatment were all positively associated with reliable recovery rates (table 2). Mean waiting time to enter treatment, the percentage of appointments that were missed, and the social deprivation of a CCG were negatively associated with reliable recovery rates (table 2). Mean waiting time to enter treatment, percentage of appointments missed, and percentage of referred patients who received a course of treatment were also significant predictors of reliable improvement in the multiple regression model (figure; other plots are shown in the appendix).

**Figure:**
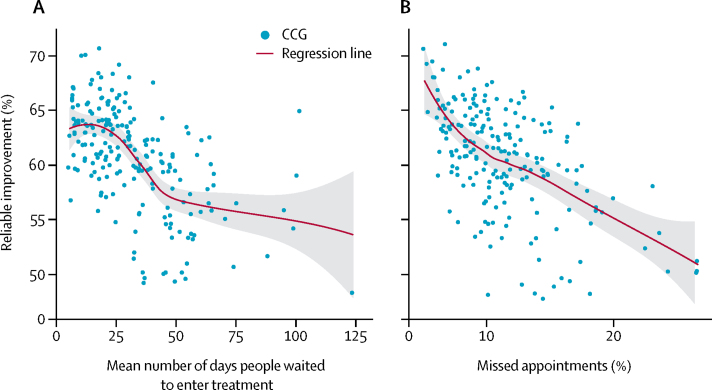
Reliable improvement of patients in relation to time waited to start treatment in a CCG (A) and missed appointments (B) Each blue dot represents predicted percentage of patients who reliably improve in a particular CCG, based on the predictor values recorded in that CCG and the effect sizes derived from the β regression. Therefore, although each graph models expected values in reference to only one of the predictors, the plotted results are a function of all six of them, accounting for the non-linear trends. Red lines were smoothed with the LOESS method, and the dark grey areas around them represent the SE around these line estimates. CCG=clinical commissioning group.

**Table 2:** Predictors of CCG performance in 2014–15

	**Reliable recovery**						**Reliable improvement**					
	Single regression			Multiple regression			Single regression			Multiple regression		
	OR (95% CI)	p value	Additive OR	OR (95% CI)	p value	Additive OR	OR (95% CI)	p value	Additive OR	OR (95% CI)	p value	Additive OR
Patients with problem descriptor completeness (%)	1·002 (1·001–1·004)	0·0015	1·221 (1·105–1·490)	1·002 (1·000–1·003)	0·0081	1·221 (1·000–1·349)	1·003 (1·001–1·004)	0·0003	1·349 (1·105–1·491)	1·001 (0·999–1·002)	0·269	1·105 (0·905–1·221)
Patients who enter treatment and receive a course of treatment (%)	1·009 (1·006–1·012)	<0·0001	1·896 (1·532–2·344)	1·004 (1·001–1·007)	0·016	1·330 (1·074–1·646)	1·014 (1·012–1·017)	<0·0001	2·698 (2·344–3·332)	1·009 (1·006–1·012)	<0·0001	1·896 (1·533–2·344)
Missed appointments (%)*	1·025 (1·015–1·035)	<0·0001	1·755 (1·404–2·190)	1·014 (1·005–1·022	0·0028	1·373 (1·120–1·642)	1·031 (1·022–1·043)	<0·0001	2·005 (1·642–2·610)	1·015 (1·006–1·024)	0·0006	1·404 (1·146–1·717)
Mean number of treatment appointments	1·077 (1·033–1·122)	0·0005	1·407 (1·161–1·698)	1·041 (1·003–1·080)	0·032	1·203 (1·014–1·425)	1·106 (1·059–1·154)	<0·0001	1·590 (1·302–1·933)	1·027 (0·990–1·065)	0·162	1·130 (0·955–1·336)
Mean number of days before entering treatment*	1·004 (1·002–1·005)	0·0005	1·598 (1·264–1·796)	1·002 (1·001–1·004)	0·015	1·264 (1·125–1·598)	1·006 (1·004–1·008)	<0·0001	2·018 (1·598–2·548)	1·003 (1·002–1·005)	0·0001	1·421 (1·264–1·796)
Index of Multiple Deprivation*	1·014 (1·010–1·019)	<0·0001	1·783 (1·513–2·188)	1·009 (1·005–1·013)	<0·0001	1·452 (1·231–1·711)	1·010 (1·005–1·015)	0·0008	1·513 (1·231–1·858)	1·002 (0·998–1·006)	0·296	1·087 (0·920–1·283)

ORs were derived by exponentiation of the β regression coefficients, and represent change in the probability of recovery or improvement with a unit increase in the predictor. Additive ORs represent ORs of recovering in the CCGs with the highest value compared with the lowest value of the given parameter. All results were taken from the model where the two CCGs identified by high Cook's Distance statistics were excluded from the analysis. OR=odds ratio. CCG=clinical commissioning group.

* Where the reciprocal of the OR originally derived from the model is given.

Table 2 shows additive ORs that estimate the changes in reliable improvement and recovery that might be achievable if the CCG with the lowest score for a particular predictor were to match the highest-scoring CCG. The analysis reveals considerable potential for improvement in the outcomes of some IAPT services. For example, if the IAPT service with the lowest percentage of patients being offered a course of treatment were to increase the proportion of people having treatment to the level of the most treatment-oriented service, recovery rates would increase by 33% and reliable improvement rates would increase by 90% (table 2).

All six predictors were individually associated with change in reliable improvement and recovery between 2014–15 and 2015–16 (appendix). In a multiple regression model, CCGs with the largest decreases in missed appointments and waiting times, and with the largest increases in problem descriptor completeness and percentage of referred patients who had a course of therapy, were also the CCGs that showed the largest increases in reliable improvement rates (table 3). Similar associations were noted for changes in reliable recovery, although fewer predictors were significant (table 3). Although social deprivation scores did not change between 2014–15 and 2015–16, CCGs with low amounts of social deprivation showed larger improvements in reliable recovery rates between 2014–15 and 2015–16 than CCGs with high amounts of social deprivation (table 3). Overall, the predictors we investigated explained a variance of 33% for change in reliable improvement and 22% for recovery rates.

**Table 3:** Predictors of change in CCG performance between 2014–15 and 2015–16

	**Reliable recovery**						**Reliable improvement**					
	Single regressions			Multiple regression			Single regressions			Multiple regression		
	β (SE)	p value	R^2^	β (SE)	p value	Partial correlation	β (SE)	p value	R^2^	β (SE)	p value	Partial correlation
Patients with problem descriptor completeness (%)	0·051 (0·017)	0·0027	0·039	0·041 (0·016)	0·011	0·137 (p=0·056)	0·055 (0·018)	0·0023	0·045	0·036 (0·015)	0·016	0·142 (p=0·047)
Patients who enter treatment and receive a course of treatment (%)	0·134 (0·034)	0·0002	0·069	0·046 (0·039)	0·231	0·033 (p=0·64)	0·272 (0·033)	<0·0001	0·249	0·187 (0·037)	<0·0001	0·248 (p=0·0004)
Missed appointments (%)*	−0·609 (0·115)	<0·0001	0·123	−0·503 (0·116)	0·0002	−0·268 (p=0·0001)	−0·761	<0·0001	0·171	−0·420 (0·113)	0·0003	−0·274 (p=0·0001)
Mean number of treatment appointments	1·261 (0·514)	0·015	0·029	0·447 (0·488)	0·361	0·066 (p=0·36)	1·935 (0·536)	0·0004	0·057	0·553 (0·467)	0·238	0·068 (p=0·343)
Mean number of days before entering treatment*	−0·053 (0·021)	0·012	0·030	−0·036 (0·021)	0·080	−0·106 (p=0·138)	−0·096 (0·022)	0·0002	0·088	−0·046 (0·020)	0·022	−0·147 (p=0·040)
Index of Multiple Deprivation	0·258 (0·077)	0·0009	0·054	0·196 (0·069)	0·0051	0·204 (p=0·0040)	0·109 (0·083)	0·190	0·009	<–0·001 (0·066)	0·996	0·002 (p=0·974)

All results were taken from the model where the one CCG identified by high Cook's Distance statistics was excluded from analyses. CCG=clinical commissioning group.

* Where the reciprocal of the OR originally derived from the model is given.

As in 2014–15, all six predictors in 2015–16 were significantly associated with both reliable improvement and reliable recovery in single regression models. β regressions with multiple predictors showed that most predictors of CCGs IAPT outcomes in 2014–15 continued to be significant predictors in 2015–16 (table 4).

**Table 4:** Predictors of CCG performance in 2015–16

	**Reliable recovery**						**Reliable improvement**					
	Single regressions			Multiple regression			Single regressions			Multiple regression		
	OR (95% CI)	p value	Additive OR	OR (95% CI)	p value	Additive OR	OR (95% CI)	p value	Additive OR	OR (95% CI)	p value	Additive OR
Patients with problem descriptor completeness (%)	1·003 (1·002–1·004)	<0·0001	1·221 (1·349–1·491)	1·001 (1·000–1·002)	0·083	1·105 (1·000–1·349)	1·003 (1·002–1·004)	0·0003	1·349 (1·221–1·491)	1·001 (1·000–1·002)	0·110	1·105 (1·000–1·221)
Patients entering treatment who receive a course of treatment (%)	1·006 (1·003–1·008)	0·0004	1·447 (1·203–1·636)	1·001 (0·999–1·002)	0·478	1·064 (0·940–1·131)	1·010 (1·007–1·013)	<0·0001	1·849 (1·538–2·220)	1·006 (1·003–1·008)	<0·0001	1·447 (1·203–1·636)
Missed appointments (%)*	1·027 (1·018–1·034)	<0·0001	1·844 (1·506–2·155)	1·017 (1·011–1·025)	<0·0001	1·473 (1·286–1·763)	1·032 (1·024–1·040)	<0·0001	2·062 (1·724–2·462)	1·021 (1·014–1·027)	<0·0001	1·612 (1·376–1·844)
Mean number of treatment appointments	1·075 (1·036–1·116)	0·0001	1·398 (1·178–1·662)	1·017 (0·987–1·048)	0·276	1·081 (0·941–1·242)	1·085 (1·042–1·129)	0·0008	1·459 (1·210–1·754)	1·011 (0·978–1·046)	0·508	1·052 (0·902–1·231)
Mean number of days before entering treatment*	1·004 (1·003–1·005)	<0·0001	1·703 (1·491–1·945)	1·002 (1·001–1·004)	0·0006	1·305 (1·143–1·703)	1·004 (1·003–1·006)	<0·0001	1·703 (1·491–1·703)	1·002 (1·001–1·004)	0·0002	1·305 (1·143–1·703)
Index of Multiple Deprivation*	1·013 (1·010–1·017)	<0·0001	1·711 (1·513–2·016)	1·010 (1·007–1·013)	<0·0001	1·513 (1·337–1·711)	1·009 (1·005–1·013)	0·0002	1·452 (1·231–1·711)	1·004 (1·001–1·007)	0·018	1·181 (1·042–1·337)

ORs were derived by exponentiation of the β regression coefficients, and represent change in the probability of recovery or improvement with a unit increase in the predictor. Additive ORs represent ORs of recovering in the CCGs with the highest value compared with the lowest value of the given parameter. All estimates were taken from the model with the CCGs identified by Cook's Distance statistics excluded. OR=odds ratio. CCG=clinical commissioning group.

* Where the reciprocal of the OR originally derived from the model is given.

The 2015–16 dataset includes some potential predictors that were not available in 2014–15. Stepped care is a key organising feature of IAPT. Simple regressions showed that CCGs in which a larger proportion of patients had low-intensity treatment only had low reliable improvement and recovery rates, whereas those CCGs with high proportions of patients who had both low-intensity and high-intensity interventions (stepped care) in their course of treatment had the highest reliable improvement and recovery rates (appendix). The proportion of patients with low-intensity and high-intensity treatment was also a significant additional predictor in the multiple regression model (appendix). To investigate whether differences between CCGs in the initial severity of patients' symptoms might have affected our findings, we ran additional multiple regressions that included mean pretreatment depression (PHQ-9) and anxiety (GAD-7) scores as additional predictors. The predictors that were significant in table 4 were significant for the additional multiple regressions analysis, and pretreatment symptom severity did not emerge as an additional predictor (appendix).

## Discussion

In this Article, our analyses suggest that looking at the way in which therapy services are implemented might also be important for the improvement of clinical outcomes. Traditionally, researchers interested in the improvement of mental health outcomes have largely focused on trying to develop new and more effective therapies, and this work has led to major advances^18^, ^19^ in psychological therapies. However, the 209 IAPT services included in the national dataset all aim to implement NICE-recommended psychological therapies^2^, ^3^ for depression and anxiety disorders using a stepped-care model with therapists who have been through a national training programme.^1^ Although the overall outcomes are broadly in line with expectation from clinical trials,^19^ the performance of individual services varies greatly.

Key aspects of the way a service is organised predict a substantial amount of this variability, both within year and when modelling changes between years. The organisational factors identified make clinical sense. Services that are better able to identify the problems they are treating (problem descriptor completeness) are presumably more likely to give the right NICE-recommended treatment. Short waits between referral and treatment might ensure patients remain enthusiastic about engaging with treatment. A high average dose of treatment (number of sessions) is likely to improve outcomes, as is consistency of attendance (low percentage of missed appointments), and a service that is predominantly focused on treatment, rather than only assessment (high percentage of patients entering treatment). Focusing on treatment is reminiscent of the positive association in surgery between postoperative outcome and the volume of operations undertaken by hospitals and individual surgeons.^20^

The range of IAPT data that is placed in the public domain on NHS Digital and Public Health England's websites is constantly expanding. Over time it will enable interested parties to explore the importance of new variables, and study how the effect of previously studied variables changes as services work to improve the way they are organised. For the first time, NHS Digital's latest annual report^4^ (October, 2016) includes CCG-level data on how stepped care is implemented. When we included this information in our model we found that services in which a high proportion of patients had some high-intensity therapy in their course of treatment had significantly better outcomes than those with a low proportion of patients. This outcome suggests services should make full use of stepped care, with patients who do not recover after low-intensity intervention (such as guided self-help) being given the opportunity to be stepped up to high-intensity intervention (traditional face-to-face therapy) rather than simply be discharged. A similar finding emerged from analysis of data from the first year of the IAPT programme, when only 32 services were available.^21^ Future IAPT reports are likely to include funding information, which could be included in statistical models.

In line with previous research,^22^ we found that social deprivation is a significant predictor of outcome. Although acting on social deprivation is a matter for local and national policy and economic development, some of its effects on outcome could possibly be mitigated by ensuring that IAPT services in socially deprived areas are of high quality and adequately funded. Consistent with this, we found that the effect sizes for social deprivation as a predictor of outcome were about halved in the multiple regressions compared with the single regressions.

IAPT is a rare example of consistent outcome monitoring and reporting in routine mental health services. Initially, many practitioners were sceptical about the feasibility of obtaining outcome data from almost all patients treated by IAPT services. This scepticism was understandable because previous attempts that were based on data collection at only the start and end of treatment were associated with high rates of missing data for after treatment.^7^ Furthermore, evidence that people who failed to provide data after treatment tended to have a poorer clinical outcome was worrying.^23^ IAPT manages to obtain an end of treatment symptom measure for almost everyone (98%) who has a course of treatment by the simple manoeuvre of asking patients to complete symptom measures every time they are seen.

Now that the possibility to obtain more or less complete outcome data is clear, and that these data can be used to identify potential ways to improve patients' outcomes, we hope mental health services elsewhere in the UK and other countries will consider adopting a similar approach to data collection and reporting. In England the IAPT dataset has greatly improved public transparency about the mental health outcomes associated with routinely delivered psychological therapies. Patients can see what their local service offers and the outcomes it achieves. Commissioners and clinicians working in the services can now benchmark their service against others and consider the development of collaborative networks in which services come together to discuss common problems and learn from each other's solutions.

Our study has some limitations. Because patient-level data were not available, we could not simultaneously estimate the effects on outcome of both patient-level variables (such as initial symptom severity, sex, age, and anxiety measures) and service organisation factors. However, we were able to show that the severity of clinical problems that services treat varied very little and that this omission cannot explain our findings. Outcome assessment was restricted to patient self-report. Although our analyses illustrate potential gains achievable in the worst-performing CCGs by bringing their organisational characteristics to the level shown by the best-performing ones, we acknowledge that these are predictions that will need to be further tested.

Before IAPT, little was known about the outcomes achieved in routine mental health services. The session-by-session outcome monitoring system used in IAPT enables the services to collect outcome data from almost everyone. Publication of this data is improving public transparency and also allows analyses to be done that help us to understand, and hopefully reduce, local variability in mental health outcomes.
