# Supplementary material for: Transparency about the outcomes of mental health services (IAPT approach): an analysis of public data
Source: Lancet. 2018 Feb 17;391(10121):679–86. doi: 10.1016/S0140-6736(17)32133-5 (PMC5820411; doi:10.1016/S0140-6736(17)32133-5)
Supplement: Supplementary appendix [file mmc1.pdf]

THE LANCET

Supplementary appendix

This appendix formed part of the original submission and has been peer reviewed. We post it as supplied by the authors.

Supplement to: Clark DM, Canvin L, Green J, Layard R, Pilling S, Janecka M. Transparency about the outcomes of mental health services (IAPT approach): an analysis of public data. *Lancet* 2017; published online Dec 7. http://dx.doi.org/10.1016/S0140-6736(17)32133-5.

SUPPLEMENTARY MATERIAL

FIGURES

Workflow of the analytical procedures applied in the study. Steps 1 and 3 refer to single-year analyses done for 2014/15 and 2015/16 periods, respectively. Step 2 refers to analyses of "delta scores", i.e. the differences in predictor and outcome levels between 2014/15 and 2015/16. Outputs of each step – either in a table or figure – are referred to in relevant boxes. Analytical aims of each step are detailed at the bottom of the diagram in green-coloured boxes.

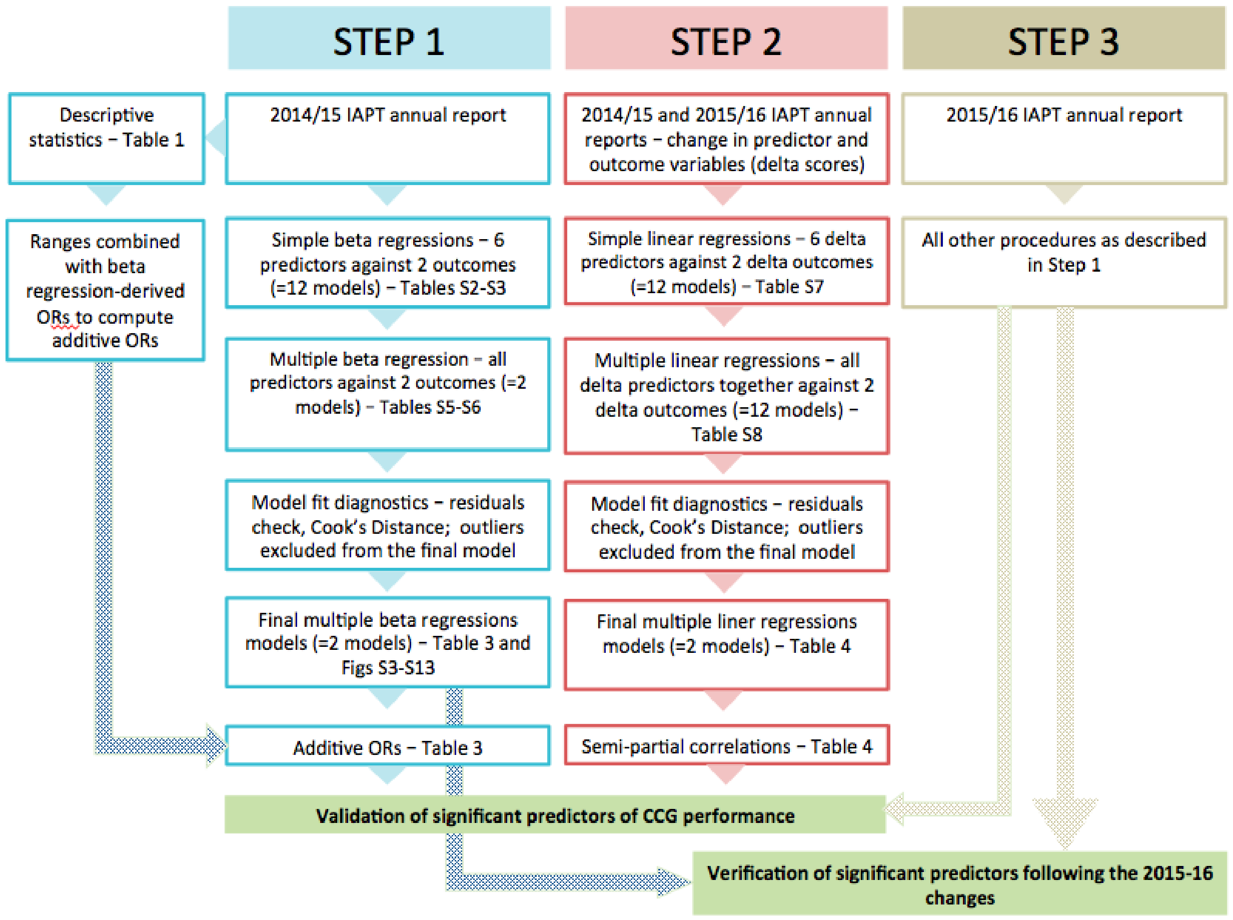

Figure S1. Schematic of the analytical workflow.

The “delta scores” of reliable improvement and recovery were normally distributed, justifying our decision to perform the analyses of change from one year to the next using linear regression.

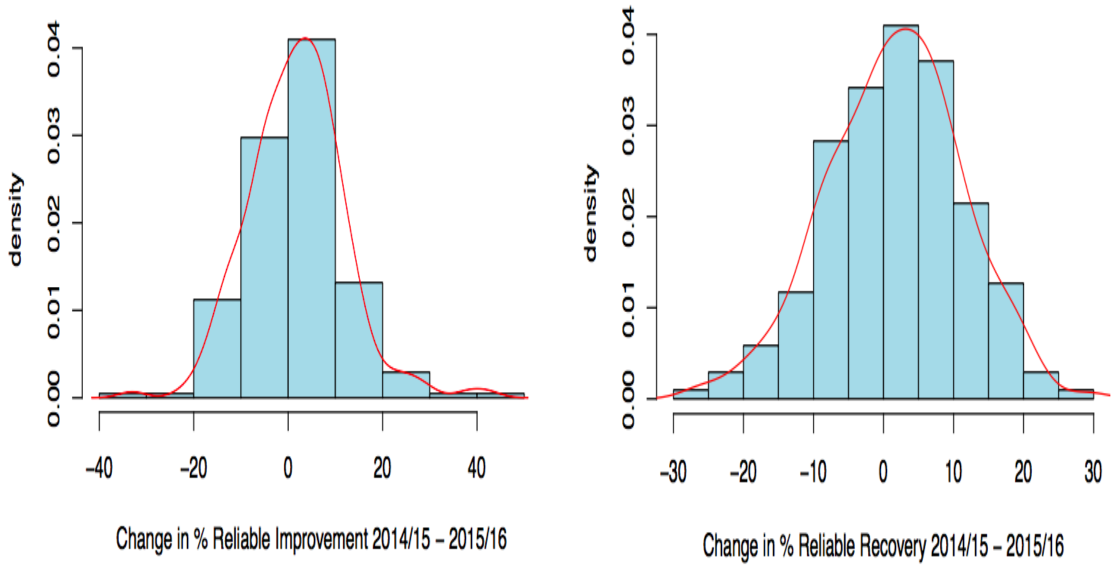

Figure S2. Distribution of rate of change of reliable improvement (A) and recovery (B) between 2014/15 and 2015/16.

Model plots

Each blue dot represents predicted percentage of patients who reliably recover / improve in a particular CCG. The predicted outcome value for each CCG is based on the predictor values recorded in that CCG and the effect sizes derived from the beta regression. Therefore, although each graph models expected values in reference to only one of the predictors, the plotted results are a function of all six of them, accounting for the non-linear trends observed below. Trend lines were smoothed using the loess method (red lines), and the dark grey areas around them represent the standard errors around the line estimates. Although the use of beta regressions necessitated use of proportions (0-1) rather than percentages (0-100), the results were converted into the latter to facilitate the graph interpretation.

Reliable Improvement

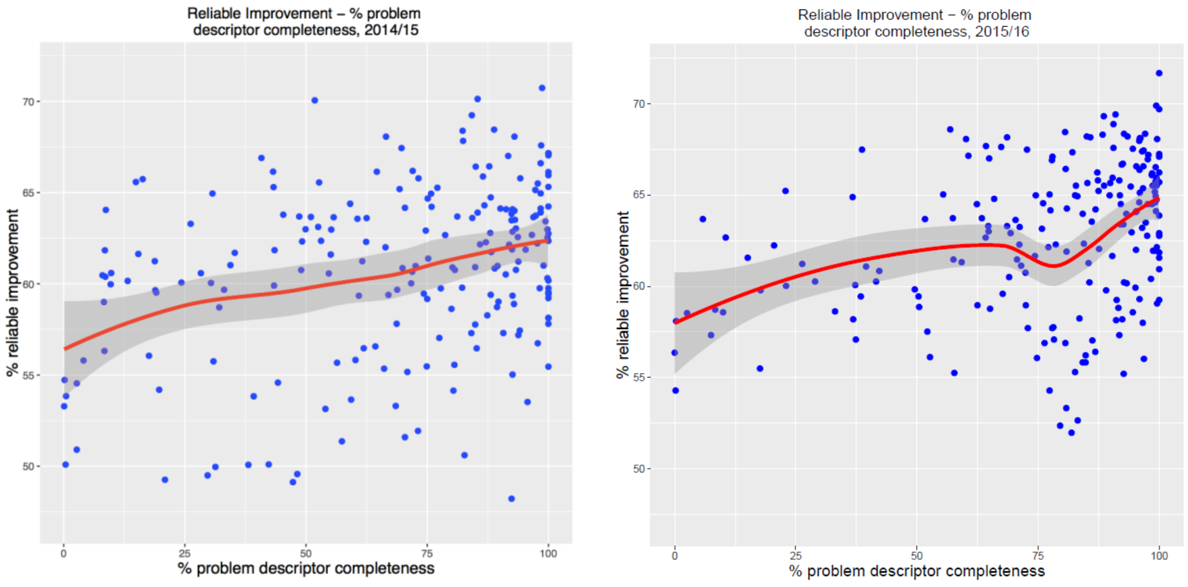

Figure S3. Percentage of patients who reliably improve in relation to percentage of patients with problem descriptor completeness in a CCG.

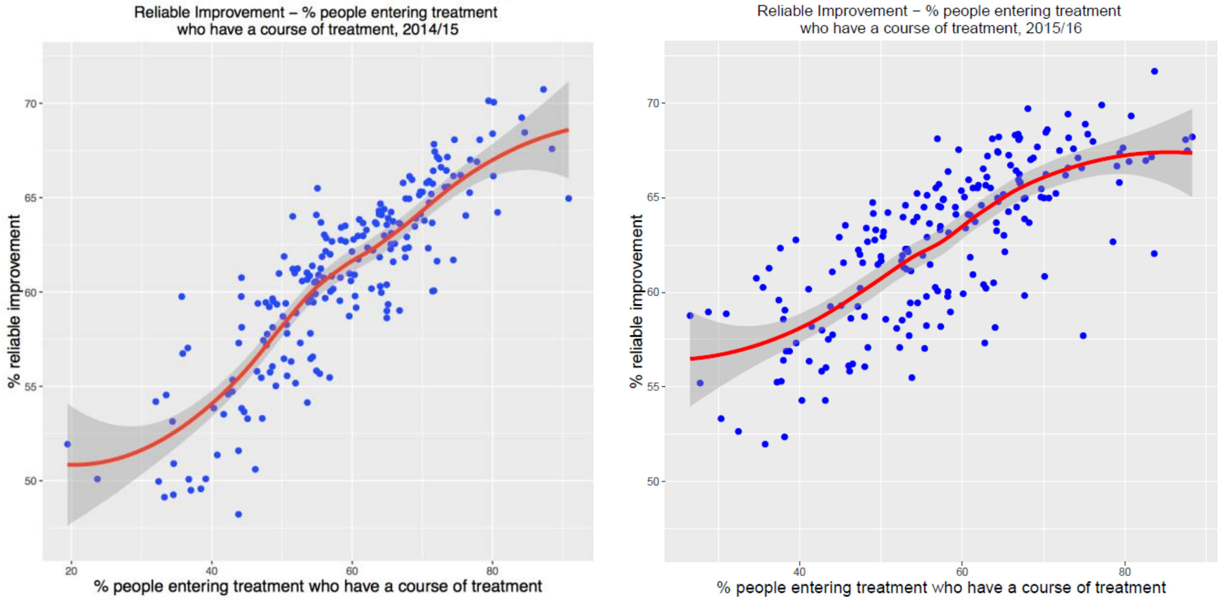

Figure S4. Percentage of patients who reliably improve in relation to people entering treatment who have a course of treatment in a CCG.

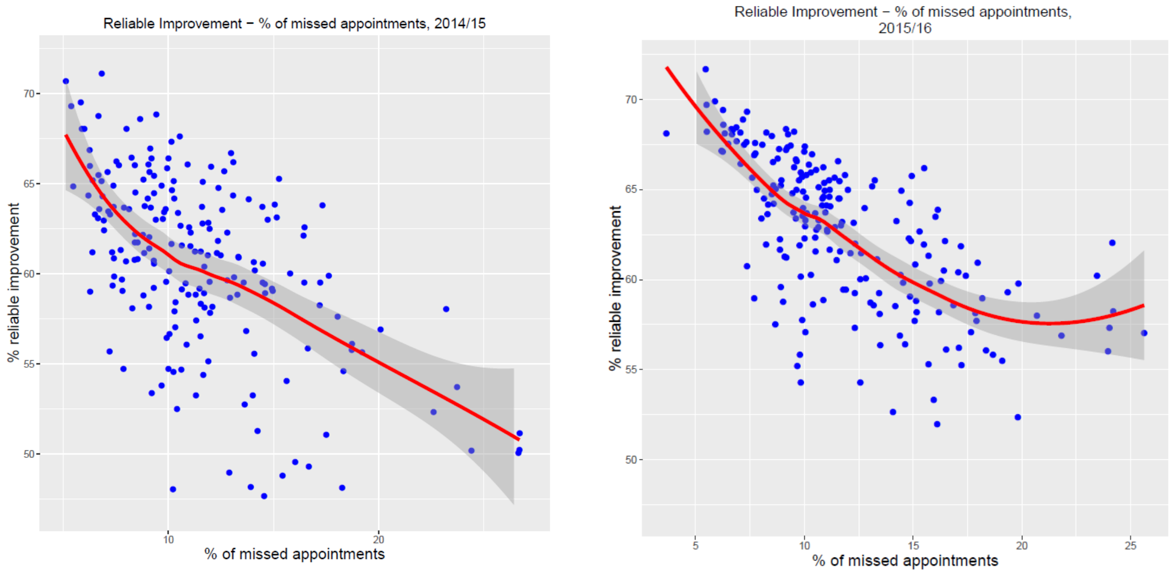

Figure S5. Percentage of patients who reliably improve in relation to percentage of missed appointments in a CCG.

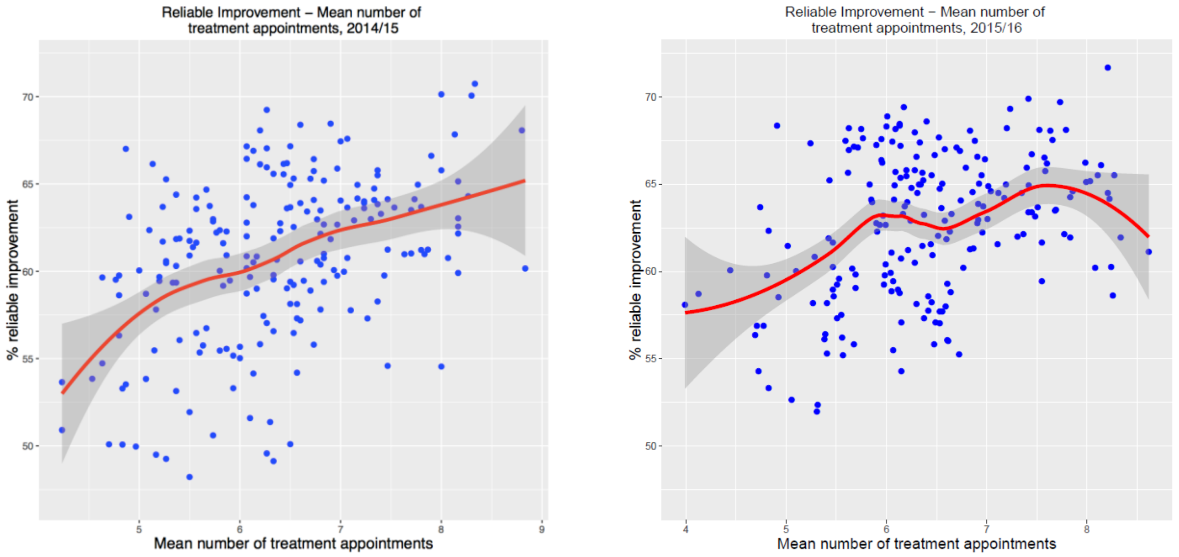

Figure S6. Percentage of patients who reliably improve in relation to mean number of treatment appointments in a CCG.

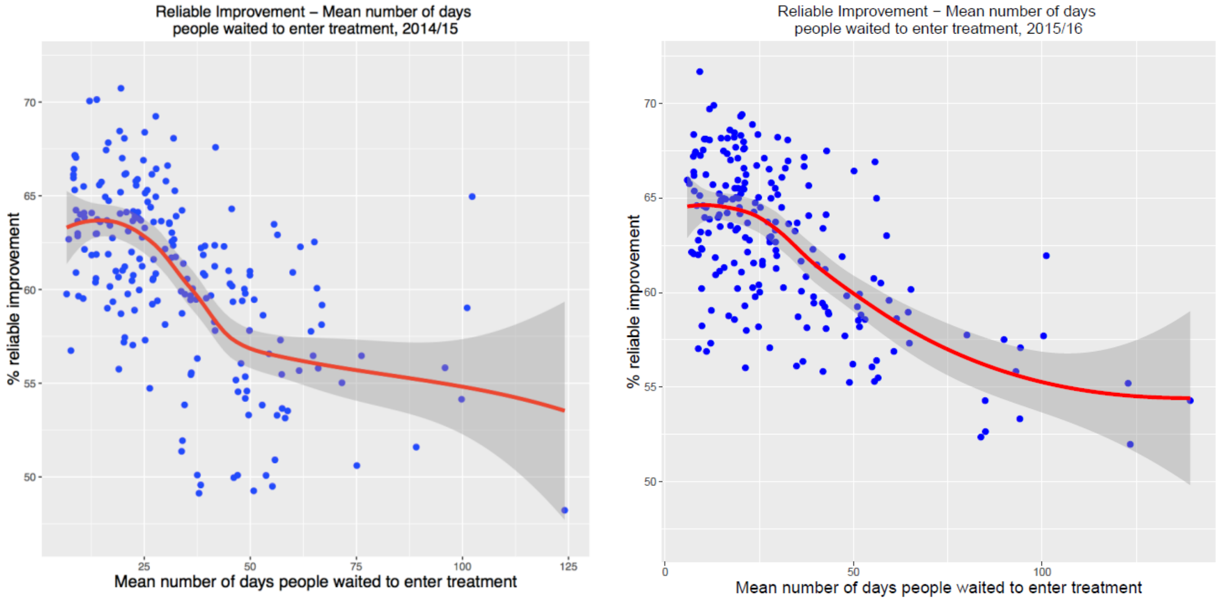

Figure S7. Percentage of patients who reliably improve in relation to mean number days people waited to enter treatment in a CCG.

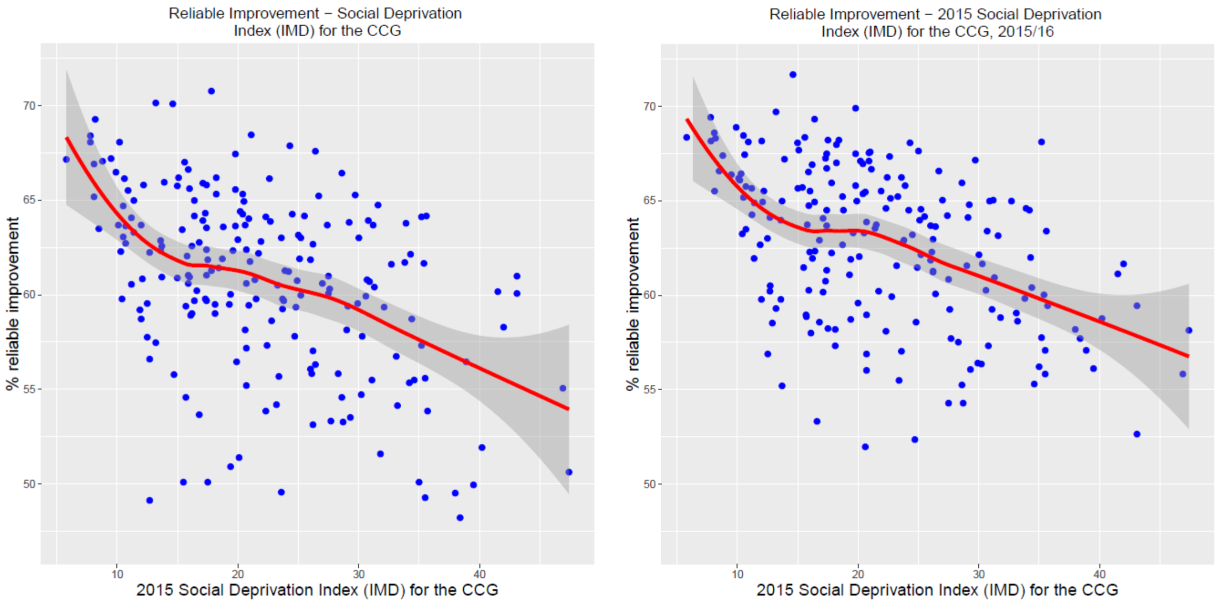

Figure S8. Percentage of patients who reliably recover in relation to social deprivation index (IMD) in a CCG.

Reliable Recovery

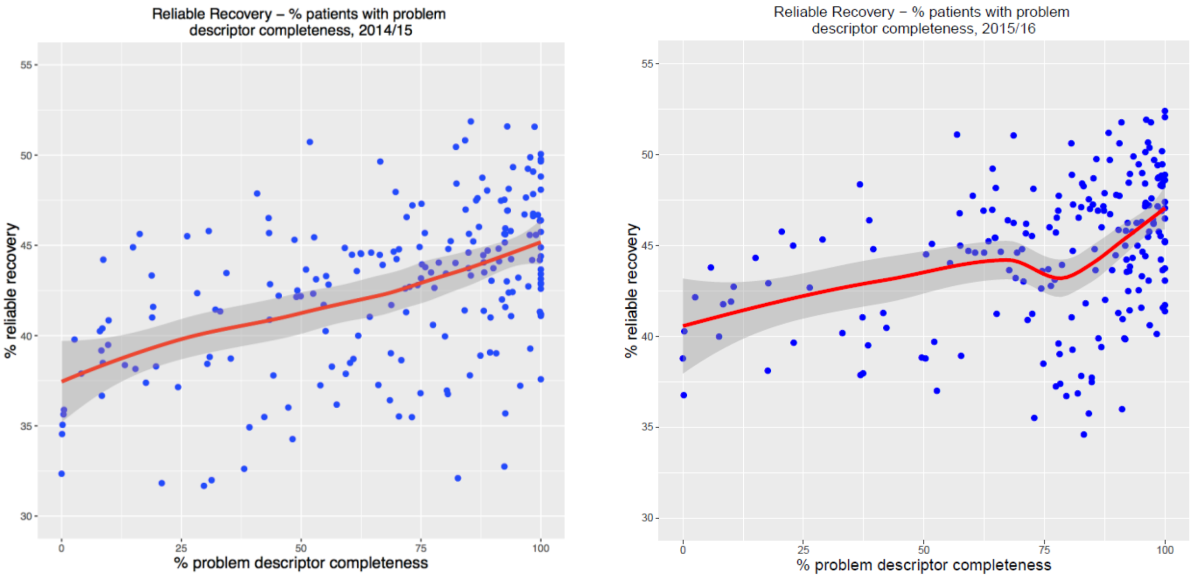

Figure S9. Percentage of patients who reliably recover in relation to percentage of patients with problem descriptor completeness in a CCG.

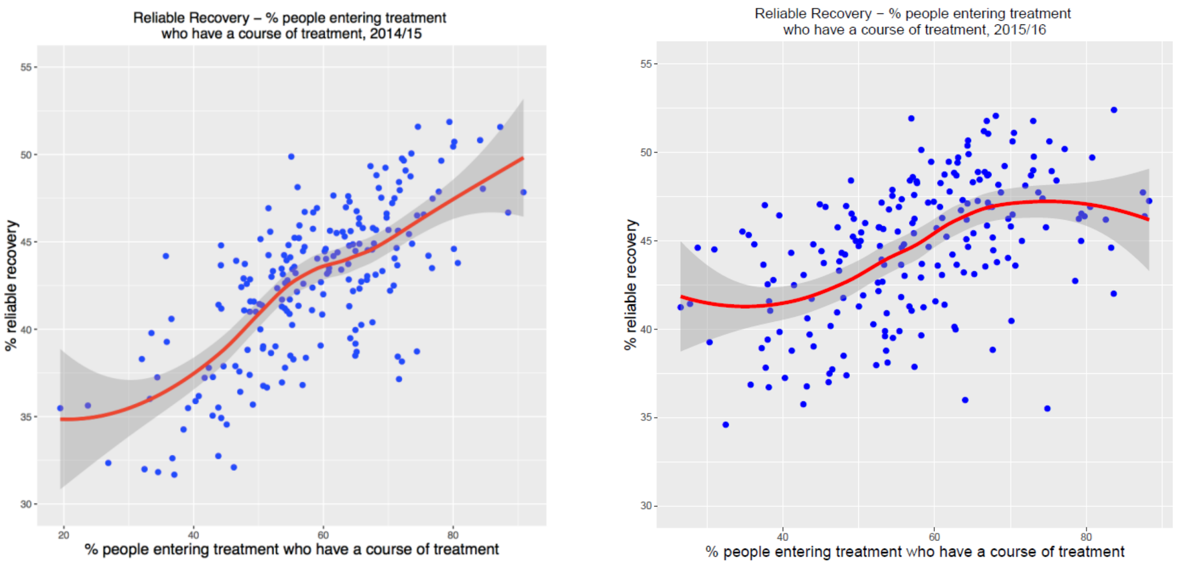

Figure S10. Percentage of patients who reliably recover in relation to percentage of people entering treatment who have a course of treatment in a CCG.

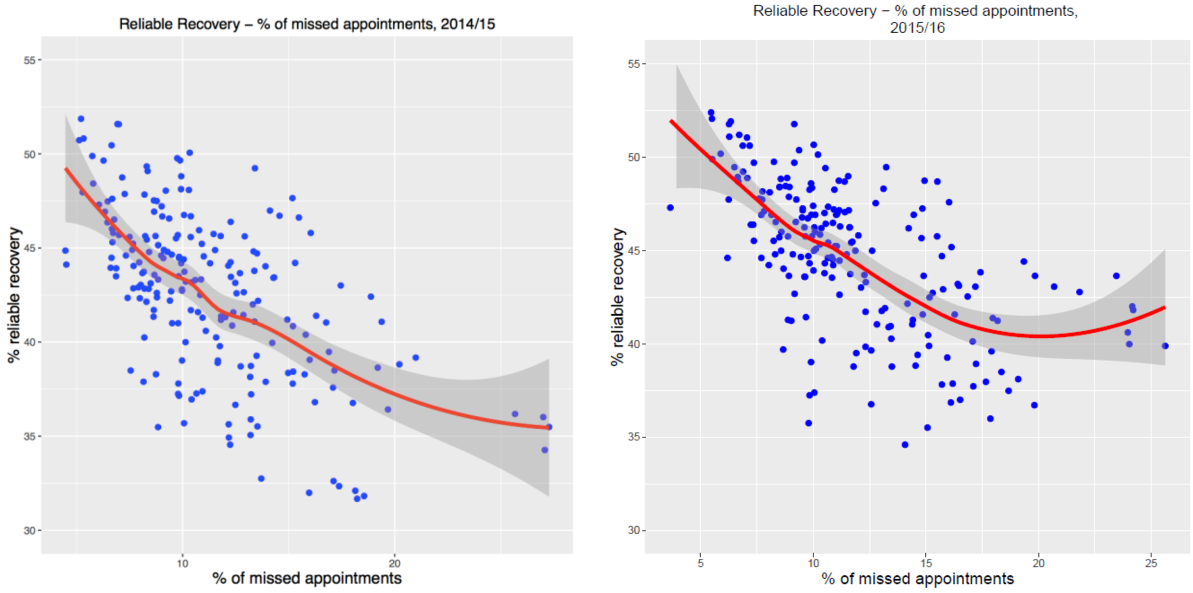

Figure S11. Percentage of patients who reliably recover in relation to percentage of missed appointments in a CCG.

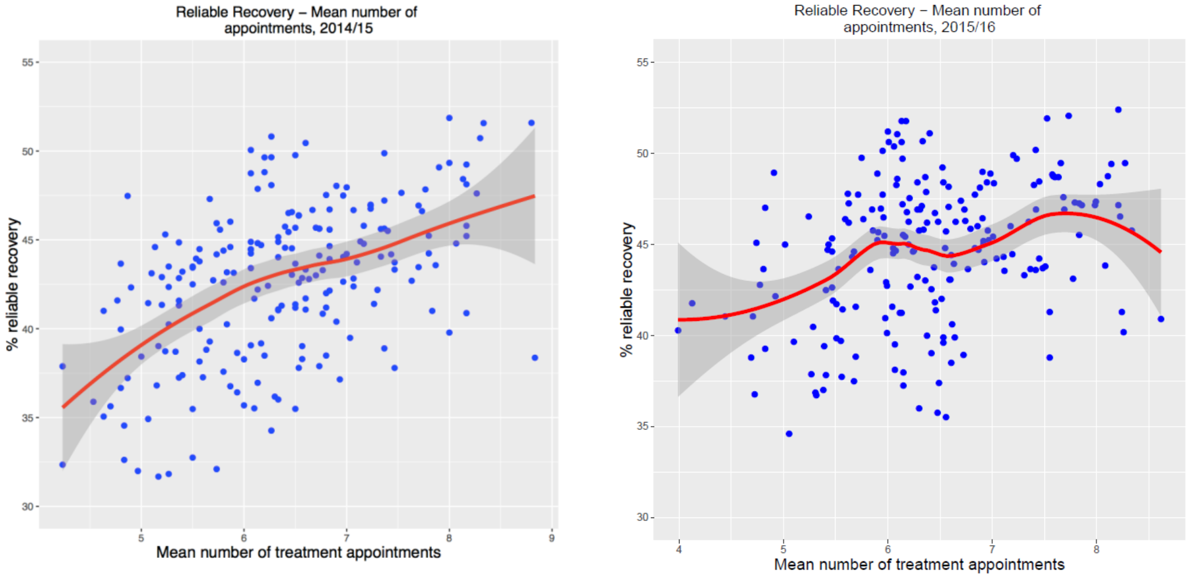

Figure S12. Percentage of patients who reliably recover in relation to mean number of treatment appointments in a CCG.

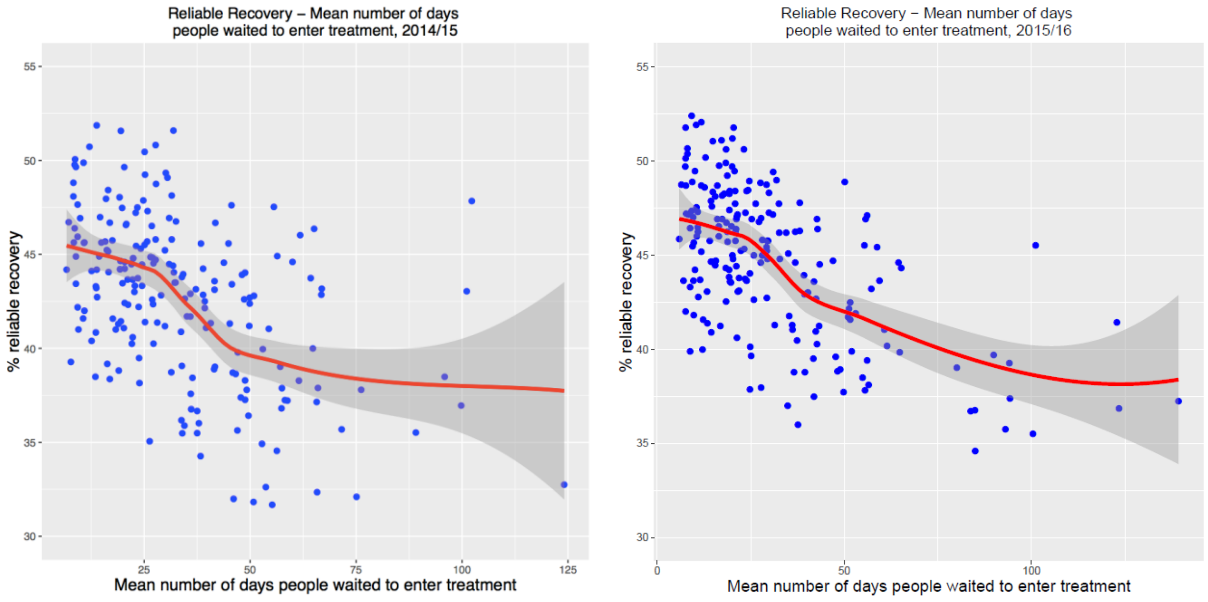

Figure S13. Percentage of patients who reliably recover in relation to mean number of days people waited for treatment in a CCG.

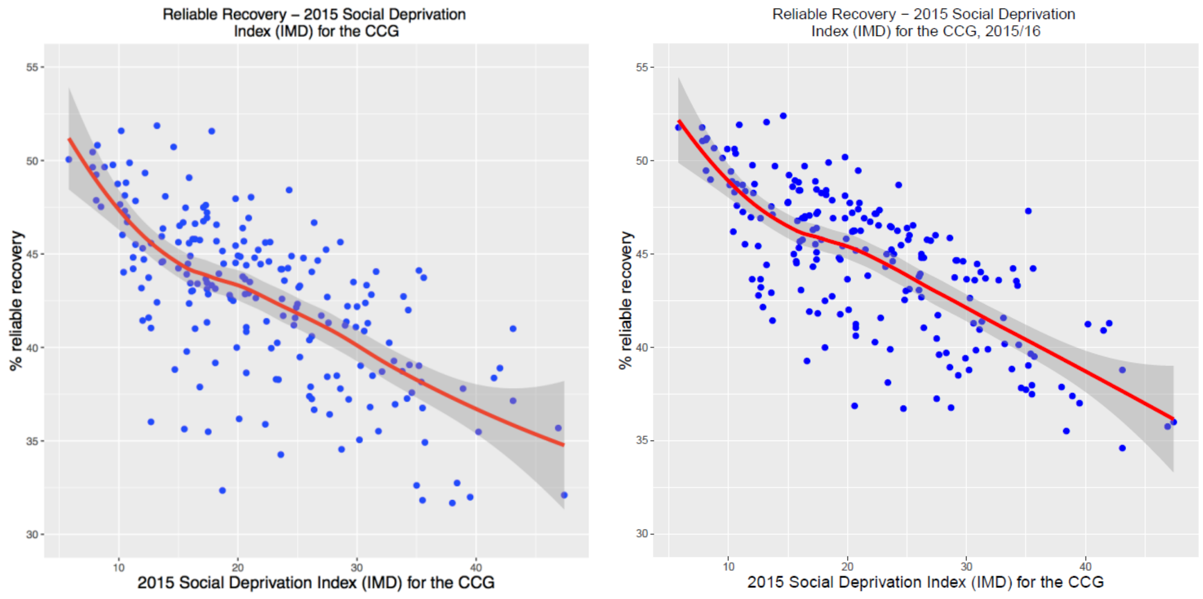

Figure S14. Percentage of patients who reliably recover in relation to social deprivation index (IMD) in a CCG.

MODEL FIT DIAGNOSTICS

The plots below (a-d) show the values of Cook's Distance (CD) statistics across the CCGs. Each horizontal line represents a single CCG, and its height reflects the influence of that CCG on the overall model fit (CD value). Spikes in the plots below indicate the CCGs with disproportionately large influence on the model parameters, likely marking outliers in the data. We inspected all CCGs with CD value of more than 0.1. Values of the predictor and outcome variables in these CCGs were compared with the sample mean, accounting for its standard deviation measure (see Table S4). Based on high levels of CD, CCGs' characteristics and residuals plots (see below) we excluded up to 2 CCGs per model, which constituted less than 1% of the total sample.

a. Reliable Recovery (2014/15)

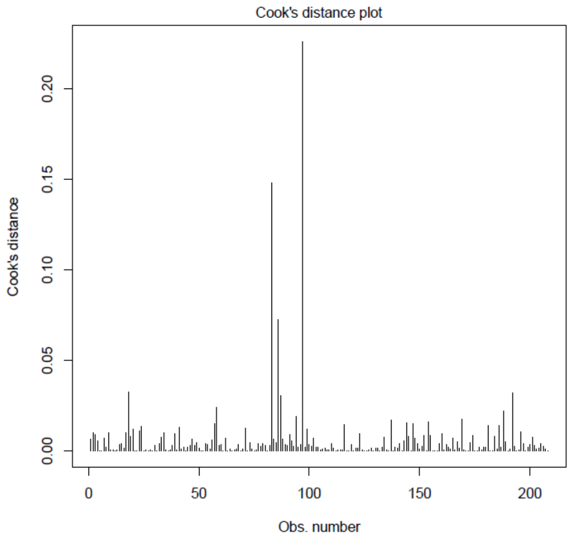

b. Reliable Improvement (2014/15)

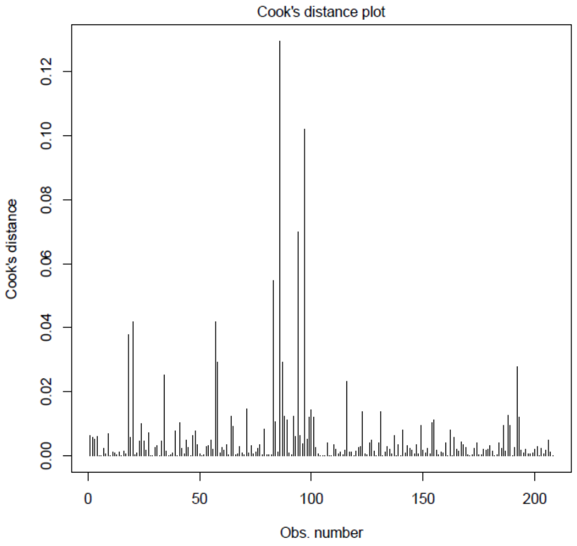

c. Reliable Recovery (2015/16)

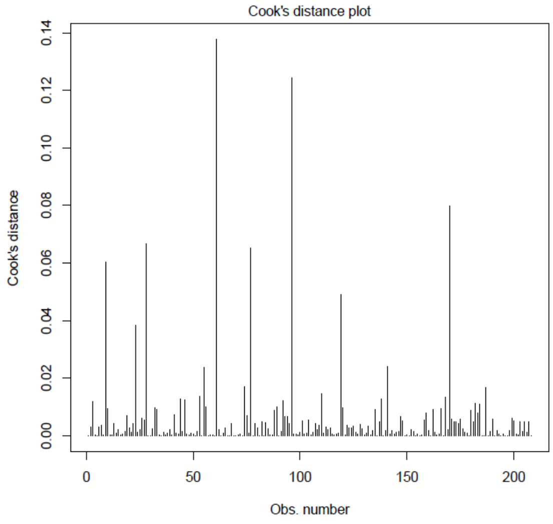

d. Reliable Improvement (2015/16)

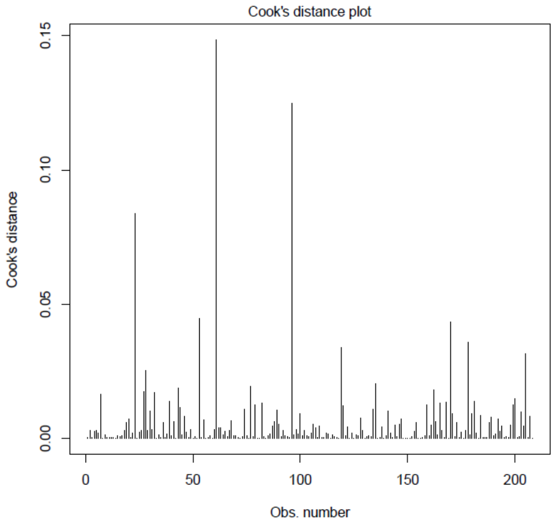

Figure S14. Plots of Cook's Distance for each CCG: (a) Reliable Recovery, 2014/15 (b) Reliable Improvement, 2014/15, (c) Reliable Recovery, 2015/16 and (d) Reliable Improvement, 2015/16. Characteristics of CCGs with Cook's Distance > 0.1 were inspected and excluded from the analyses.

The diagrams below represent half-normal probability plots of residuals for multiple regression models for rates of reliable recovery and reliable improvement, for each of the two reporting periods analysed in the current study. Such plots are often used in evaluating beta regression models, and are constructed as normal probability plots of standardized residuals, except for the value of residuals are here expressed as an absolute value (i.e. depriving the negative values of their (-) sign), allowing for comparing their magnitudes regardless of the negative/positive value. Each circle represents an absolute value of the standardized residual for a given CCG – i.e. how much its actual outcome values were departing from those predicted by the model. All values ideally should fall between the envelopes around the main line, with major departures beyond these envelopes signalling problems with the model fit. Below we present half-normal probability plots of residuals constructed before and after excluding the CCGs with particularly high influence on the model fit. These graphs support excluding the CCGs with high levels of Cook's Distance, and illustrate the goodness of model fit achieved in the current study.

a. Reliable Recovery (2014/15) – pre-exclusion

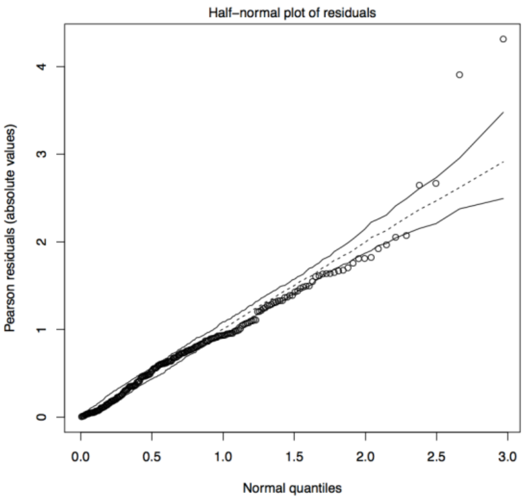

b. Reliable Recovery (2014/15) – post-exclusion

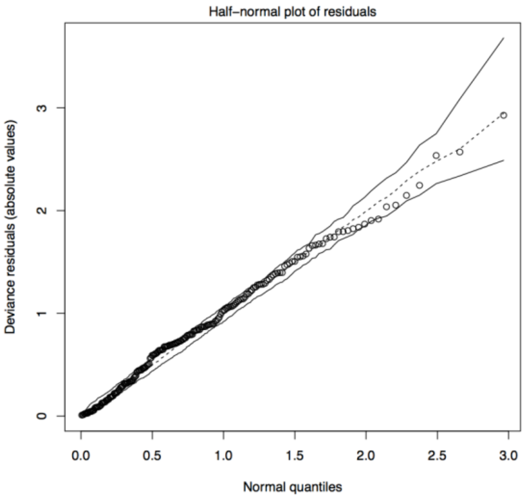

c. Reliable Recovery (2015/16) – pre-exclusion

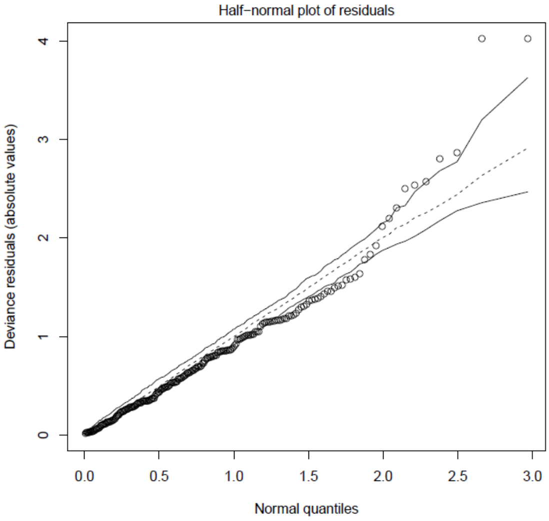

d. Reliable Recovery (2015/16) – post-exclusion

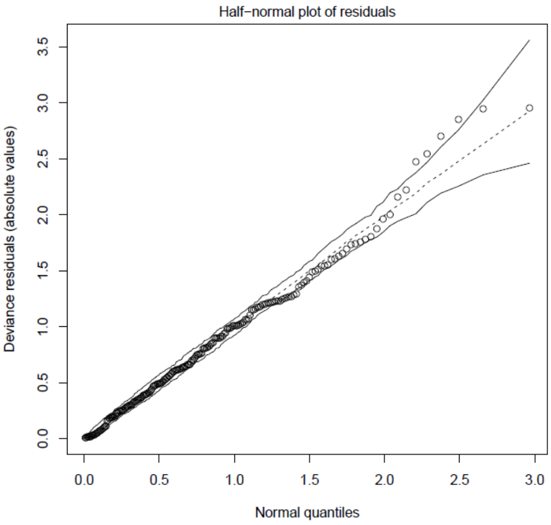

Figure S15. Half normal plot of Pearson's residuals for Reliable Recover model, (a) 2014/15, before exclusions, (b) 2014/15, after exclusions, (c) 2015/16, before exclusions, and (d) 2015/16, after exclusions of the CCGs with highest influence over the model fit.

a. Reliable Improvement (2014/15) – pre-exclusion

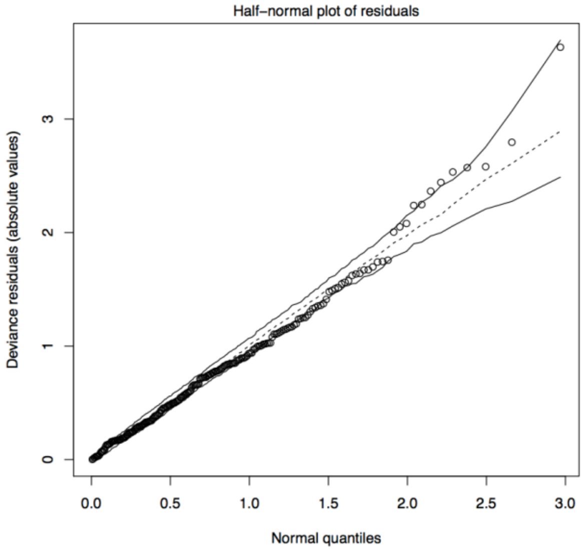

b. Reliable Improvement (2014/15) – post-exclusion

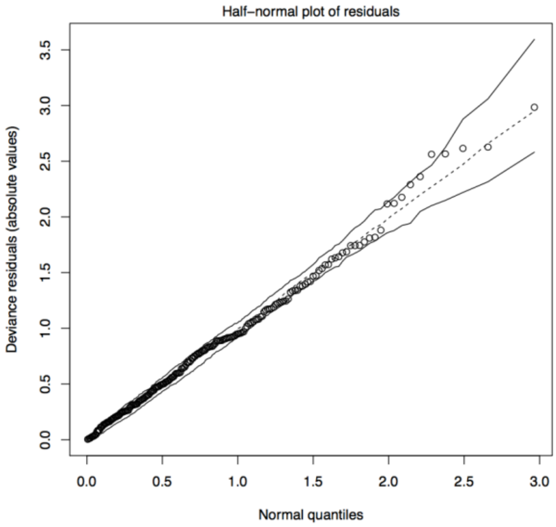

c. Reliable Improvement (2015/16) – pre-exclusion

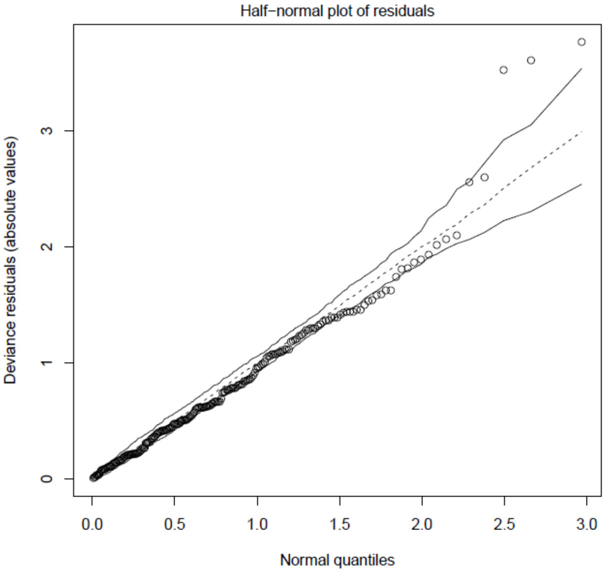

d. Reliable Improvement (2015/16) – post-exclusion

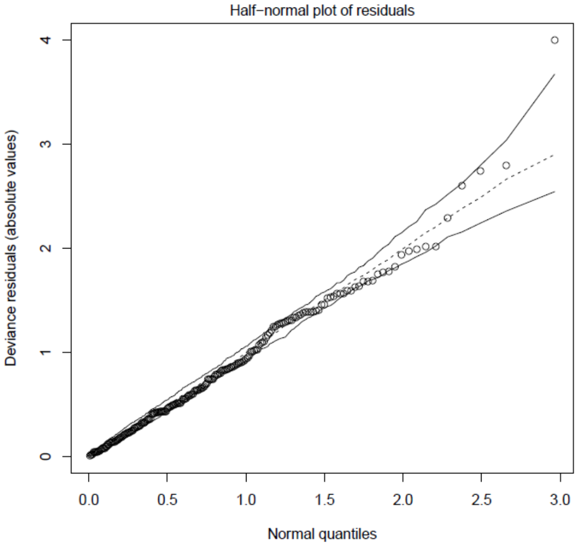

Figure S16. Half normal plot of Pearson's residuals for Reliable Improvement model before (1) for 2014/15, before (A) and after (B) excluding the two CCGs with highest influence over the model fit, and (2) for 2015/16, before (A) and after (B) excluding the two CCGs with highest influence over the model fit.

The plots below represent normal probability plots for the models of change in rates of reliable improvement and recovery from one year to the next. The standardized residuals of the linear regression is compared with the normal distribution, allowing for verifying the model assumptions. Major departures from a straight diagonal line would indicate non-normality of the distribution of residuals, and therefore violations of the model assumptions. Below we present the plots before and after excluding the CCGs with high values of Cook's Distance statistic. These plots both justify excluding the CCGs with high levels of Cook's Distance, and validate our approach to test our hypotheses using linear regression.

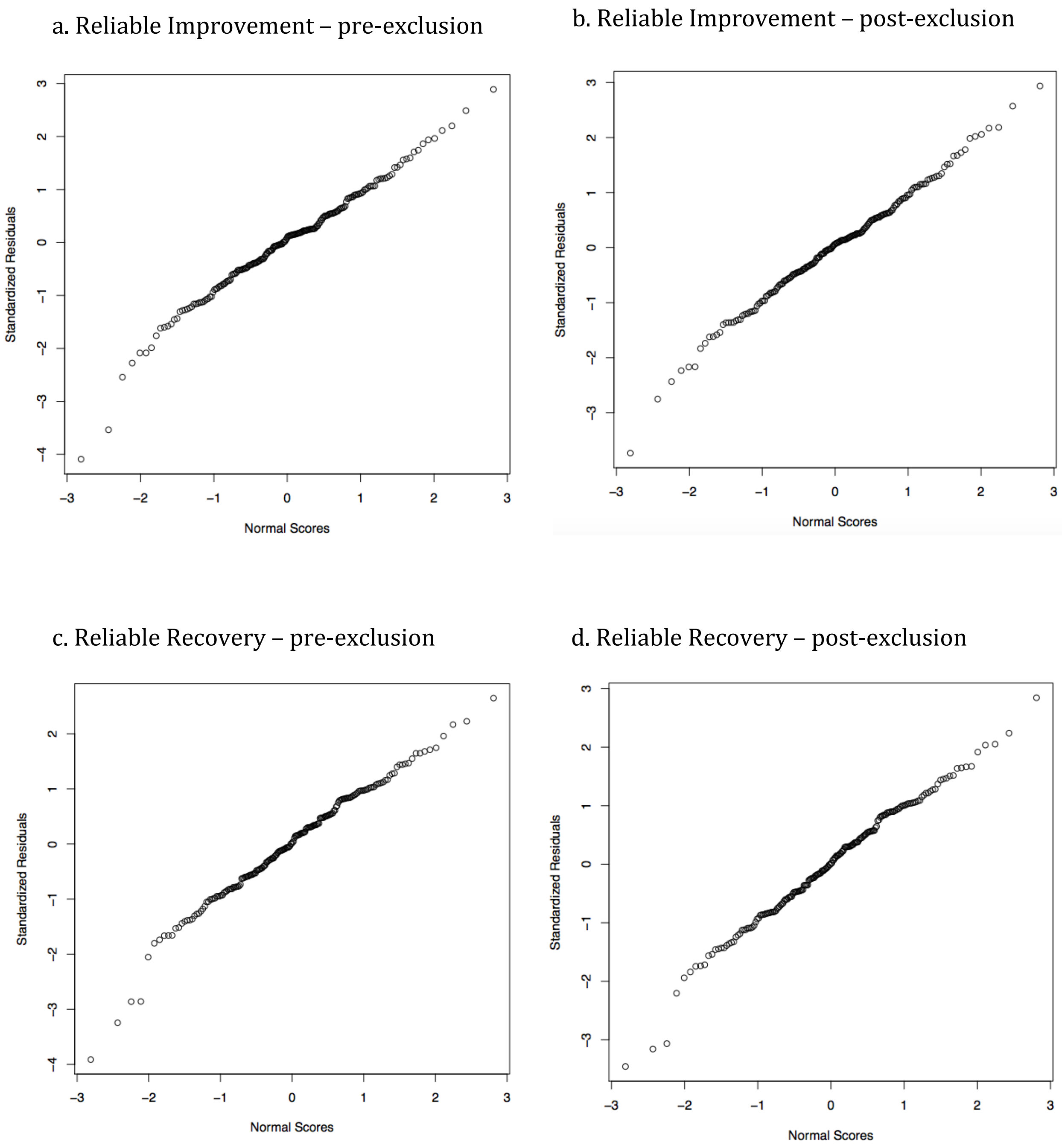

Figure S17. QQ plots of standardized residuals for models of predictors of change in CCG performance between 2014/15 and 2015/16. Reliable improvement, (a) before, and (b) after exclusions, and Reliable Recovery, (c) before, and (d) after exclusion of the one CCG with highest influence over the model fit.

TABLES

The table below provides mean change (mean "delta scores") in the predictor and outcome levels in the 10% of the CCGs that improved most (top 10%), 10% that improved least/deteriorated (bottom 10%) and the sample overall. We used these observations to explore whether year-to-year change in the levels of predictors was paralleled by change in the outcomes, which then drove our analyses to formally test these patterns. The values in the table represent difference between levels of each variable in 2014/15 and 2015/16, with positive values indicating increase, and negative values decrease over time.

	Reliable Improvement			Reliable Recovery		
	Top 10%	Sample mean	Bottom 10%	Top 10%	Sample mean	Bottom 10%
**Δ % Recovery / Improvement**	20.46 (8.14)	1.83 (10.24)	-15.32 (4.88)	17.98 (3.48)	1.54 (9.64)	-15.72 (4.60)
**% patients with problem descriptor completeness**	32.75 (34.05)	8.08 (39.50)	-11.53 (38.28)	33.31 (44.23)	8.08 (39.50)	-5.56 (35.29)
**% patients entering treatment who receive a course of treatment**	21.00 (13.66)	-0.40 (18.80)	-13.17 (21.38)	14.53 (15.98)	-0.40 (18.80)	-1.06 (24.68)
**% missed appointments**	-2.81 (5.59)	0.51 (5.59)	4.45 (5.23)	-2.62 (5.16)	0.51 (5.59)	3.43 (6.44)
**Mean number of treatment appointments**	1.05 (1.18)	0.04 (1.32)	-0.15 (1.46)	0.65 (1.33)	0.04 (1.32)	-0.56 (1.51)
**Mean number of days before entering treatment**	-27.70 (17.81)	-2.51 (31.66)	7.30 (45.76)	-22.00 (18.26)	-2.51 (31.66)	0.83 (41.24)

Table S1. Mean change and its standard deviation in the parameters used as CCG performance predictors, in the whole sample, 10% of CCGs that improved most, and 10% that improved least / deteriorated over the study period (as measured by change in rates of patients who reliably recover / improve in those CCGs).

The Table below presents characteristics of the CCGs with particularly high influence over model fit (the “spikes” visible in Fig S14. These characteristics were examined to decide whether to retain or not these data points in the final analyses.

	2014/15			
	04Y - NHS Cannock Chase CCG^{RR}	05D - NHS East Staffordshire CCG^{RI}	05V - NHS Stafford and Surrounds CCG$^{RI, RR}$	Sample mean (SD)
**% patients with problem descriptor completeness**	2.7	0	1.3	67.8 (29.9)
**% missed appointments**	12.9	17.8	17.3	11.3 (4.3)
**Mean number of treatment appointments**	4.1	4.5	5.1	6.4 (1.1)
**Mean number of days before entering treatment**	55.8	65.8	89.3	33.7 (20.6)
**IMD**	19.4	18.7	12.9	22.0 (8.7)
**% patients entering treatment who receive a course of treatment**	0.4	0.3	0.3	0.58 (0.13)
**% reliable improvement**	65.8	24.8	61.4	60.6 (7.8)
**% reliable recovery**	64.6	18.6	61.5	42.9 (7.5)
	**2014/15-2015/16 change**			
	**04Y – NHS Cannock Chase ^{RR}**	**03K – NHS North Lincolnshire^{RI}**		**Sample mean (SD)**
**Δ % patients with problem descriptor completeness**	79.4	32.93		8.1 (39.5)
**Δ % missed appointments**	-3.8	11.85		-0.4 (18.8)
**Δ Mean number of treatment appointments**	1.0	1.04		0.5 (5.6)
**Δ Mean number of days before entering treatment**	-39.3	-16.4		0.04 (1.3)
**Δ % patients entering treatment who receive a course of treatment**	44.8	13.61		-2.5 (31.7)
**Δ % reliable improvement**	-4.4	-33.0		1.5 (9.6)
**Δ % reliable recovery**	-24.9	-26.5		8.1 (39.5)
	**2015/6**			
	**04C - NHS Leicester City CCG^{RR,RI}**	**11M - NHS Gloucestershire CCG$^{RR, RI}$**		**Sample mean (SD)**
**% patients with problem descriptor completeness**	13.3	33.4		76.2 (25.7)
**% missed appointments**	13.7	24.1		12.1 (4.3)
**Mean number of treatment appointments**	5.1	5.6		6.4 (0.9)
**Mean number of days before entering treatment**	36.5	18.1		31.0 (23.3)
**IMD**	32.1	14.7		22.0 (8.7)
**% patients entering treatment who receive a course of treatment**	75.3	53.9		57.7 (12.9)
**% reliable improvement**	41.4	35.4		62.5 (6.6)
**% reliable recovery**	20.4	21.2		44.4 (6.3)

Table S2. Characteristics of the CCGs with largest influence over the model fit for Reliable Recovery (RR superscript) or Reliable Improvement (RI superscript), as estimated by the Cook’s Distance statistic, 2015 and 2016 data.

The tables below present results from the multiple beta regressions investigating the predictors of rates of reliable improvement and recovery across the CCGs. These analyses mimic those presented in Tables 2 and 4 of the original article, but rely on the sample without any exclusions. Although we could justify not including certain CCGs in the final analyses – by inspecting their characteristics, as well as reliably showing that excluding them led to improvements in model fit – for the transparency reasons we decided to present them for others to explore. The additive ORs are not presented in the tables below.

	**Reliable Improvement**					
	**2014/15**			**2015/16**		
	**Estimate (SE)**	**p**	**OR (95% CIs)**	**Estimate (SE)**	**p**	**OR (95% CIs)**
**% patients with problem descriptor completeness**	0.001 (0.001)	0.239	1.001 (0.995-1.002)	0.002 (0.001)	0.016	1.002 (1.000-1.003)
**% patients entering treatment who receive a course of treatment**	0.009 (0.002)	<0.001 (4.4E-8)	1.009 (1.006-1.012)	0.005 (0.001)	<0.001 ($2.09E^{-4}$)	1.005 (1.002-1.008)
**% missed appointments[l]**	-0.016 (0.005)	<0.001 (9.05E-4)	1.016 (1.006-1.026)	-0.024 (0.004)	<0.001 ($7.32E^{-11}$)	1.024 (1.016-1.031)
**Mean number of treatment appointments**	0.030 (0.020)	0.127	1.030 (0.992-1.070)	0.016 (0.018)	0.369	1.017 (0.981-1.054)
**Mean number of days before entering treatment[l]**	-0.003 (0.001)	<0.001 (4.18E-4)	1.003 (1.001-1.005)	-0.002 (0.001)	0.001	1.002 (1.001-1.004)
**Index of Multiple Deprivation[l]**	-0.002 (0.002)	0.301	1.002 (0.998-1.006)	-0.004 (0.002)	0.027	1.004 (1.000-1.007)

Table S3. Multiple regression coefficients for the Reliable Improvement models for 2014/15 and 2015/16. Estimates of the regression coefficients are presented with their standard errors and significance levels. Odds ratios were derived by exponentiating the regression coefficients, and represent change in the probability of recovery with a unit increase in the predictor. ([l]) indicates where the reciprocal of the OR originally derived from the model is given. All estimates were taken from the model using the full sample (no exclusions).

	Reliable Recovery					
	2014/15			2015/16		
	Estimate (SE)	p	OR (95% CIs)	Estimate (SE)	p	OR (95% CIs)
**% patients with problem descriptor completeness**	0.001 (0.001)	0.132	1.001 (1.000-1.002)	0.002 (0.001)	0.008	1.002 (1.000-1.003)
**% patients entering treatment who receive a course of treatment**	0.003 (0.002)	0.128	1.003 (0.999-1.006)	<0.001 (0.001)	0.984	1.000 (0.998-1.002)
**% missed appointments[I]**	-0.014 (0.004)	0.006	1.014 (1.004-1.023)	-0.020 (0.003)	<0.001 ($1.01E^{-9}$)	1.020 (1.014-1.025)
**Mean number of treatment appointments**	0.030 (0.020)	0.014	1.030 (0.990-1.072)	0.023 (0.017)	0.176	1.023 (0.990-1.057)
**Mean number of days before entering treatment[I]**	-0.001 (<0.001)	0.155	1.003 (0.999-1.003)	-0.002 (0.001)	<0.001 ($4.61E^{-4}$)	1.002 (1.001-1.004)
**Index of Multiple Deprivation[I]**	-0.011 (0.002)	<0.001 ($2.17E^{-6}$)	1.011 (1.006-1.015)	-0.010 (0.002)	<0.001 ($1.58E^{-9}$)	1.010 (1.007-1.013)

Table S4. Multiple regression coefficients for the Reliable Recovery models for 2014/15 and 2015/16. Estimates of the regression coefficients are presented with their standard errors and significance levels. Odds ratios were derived by exponentiating the regression coefficients, and represent change in the probability of recovery with a unit increase in the predictor. ([I]) indicates where the reciprocal of the OR originally derived from the model is given. All estimates were taken from the model using the full sample (no exclusions).

The table below present results from the multiple linear regressions investigating the predictors of change in the rates of reliable improvement and recovery from one year to the next. These analyses mimic those presented in Table 3 of the original article, but rely on the sample without any exclusions. Although we could justify not including certain CCGs in the final analyses – by inspecting their characteristics, as well as reliably showing that excluding them led to improvements in model fit – for the transparency reasons we decided to present them for others to explore.

	2014/15-2015/16 change					
	Reliable Recovery			Reliable Improvement		
	β (SE)	p	Partial correlation	β (SE)	p	Partial correlation
**% patients with problem descriptor completeness**	0.033 (0.016)	0.045	0.137 (p=0.06)	0.033 (0.016)	0.038	0.142 (p=0.047)
**% patients entering treatment who receive a course of treatment**	0.023 (0.040)	0.571	0.033 (p=0.64)	0.171 (0.038)	<0.001 ($1.36E^{-5}$)	0.248 (p<0.001)
**% missed appointments**	-0.514 (0.120)	<0.001 ($2.98E^{-5}$)	-0.268 (p<0.001)	-0.506 (0.116)	<0.001 ($1.90E^{-5}$)	-0.274 (p<0.001)
**Mean number of treatment appointments**	0.510 (0.507)	0.315	0.066 (p=0.36)	0.504 (0.487)	0.302	0.068 (p=0.343)
**Mean number of days before entering treatment**	-0.035 (0.021)	0.099	-0.106 (p=0.138)	-0.048 (0.021)	0.022	-0.147 (p=0.040)
**Index of Multiple Deprivation**	0.213 (0.072)	0.003	0.204 (p=0.004)	0.002 (0.069)	0.973	0.002 (p=0.974)

Table S5. Multiple regression coefficients of the association between change in CCG parameters used as performance predictors, and rates of reliable recovery / improvement, between 2014/15 and 2015/16. All results were taken from the model including all CCGs.

	Reliable improvement			Reliable recovery		
	Estimate (SE)	p	OR (95% CI)	Estimate (SE)	p	OR (95% CI)
**Low only**[I]	-0.004 (0.001)	<0.001	1.004 (1.002-1.006)	-0.003 (0.001)	0.010	1.003 (1.001-1.005)
**High only**[I]	-0.0002 (<0.001)	0.798	1.000 (0.998-1.002)	-0.001 (0.001)	0.086	1.001 (1.000-1.003)
**Low and high**	0.005 (0.001)	<0.001	1.005 (1.003-1.008)	0.006 (0.001)	<0.001	1.006 (1.004-1.008)

Table S6. Single regressions for treatment intensity in 2015/16 data. ([I]) indicates where the reciprocal of the OR originally derived from the model is given.

	Reliable improvement			Reliable recovery		
	Estimate (SE)	p	OR (95% CI)	Estimate (SE)	p	OR (95% CI)
**% patients with problem descriptor completeness**	0.001 (0.001)	0.041	1.001 (1.000-1.002)	0.001 (0.001)	0.017	1.001 (1.000-1.003)
**% patients entering treatment who receive a course of treatment**	0.005 (0.001)	<0.001	1.005 (1.002-1.008)	<0.001 (0.001)	0.717	1.000 (0.998-1.003)
**% missed appointments**[I]	-0.022 (0.004)	<0.001	1.022 (1.014-1.030)	-0.018 (0.004)	<0.001	1.018 (1.011-1.026)
**Mean number of treatment appointments**	0.014 (0.018)	0.455	1.014 (0.978-1.051)	0.016 (0.017)	0.352	1.016 (0.983-1.050)
**Mean number of days before entering treatment**[I]	-0.002 (0.001)	0.008	1.002 (1.001-1.004)	-0.002 (0.001)	0.014	1.002 (1.000-1.003)
**Index of Multiple Deprivation**[I]	-0.004 (0.002)	0.015	1.004 (1.001-1.008)	-0.010 (0.002)	<0.001	1.010 (1.007-1.013)
**High intensity only**	0.002 (0.001)	0.007	1.003 (1.001-1.006)	0.002 (0.001)	0.038	1.002 (1.000-1.003)
**Low and high intensity**	0.003 (0.001)	0.019	1.003 (1.001-1.005)	0.003 (0.001)	0.006	1.003 (1.001-1.006)

S7. Multiple regressions including the effects of the treatment intensity (high only, low and high) in 2015/16 data. ([I]) indicates where the reciprocal of the OR originally derived from the model is given.

CONTROLLING FOR DIFFERENCES BETWEEN CCGS IN THE INITIAL SEVERITY OF SYMPTOMS IN DEPRESSION AND ANXIETY CASES.

Unlike the 2014/15 dataset, the 2015/16 dataset includes information on the average (mean) PHQ-9 and GAD-7 scores for patients treated in each CCG's IAPT service. Inspection of the data indicates that patients who present for treatment in the difference services appear to have similarly severe symptoms at the start of treatment. The PHQ -9 is a scale that ranges from 0 to 27. The average PHQ score for depressed patients treated in the IAPT services is 17.30 with a very small standard deviation (0.74). The GAD-7 is a scale that ranges from 0 to 21. The average GAD score for patients with anxiety disorders treated in the IAPT services is 14.8 and the standard deviation is once again extremely small (0.50). In the additional analysis reported below we added average pre-treatment PHQ-9 and GAD-7 scores to the multiple regression reported in the paper. All the organizational factors that were related to outcome in our original analysis remain significant in this additional analysis.

	Reliable improvement			Reliable recovery		
	**Estimate (SE)**	**p**	**OR (95% CI)**	**Estimate (SE)**	**p**	**OR (95% CI)**
**% patients with problem descriptor completeness**	0.002 (0.001)	0.009	1.002 (1.000-1.003)	0.002 (0.001)	0.001	1.002 (1.001-1.003)
**% patients entering treatment who receive a course of treatment**	0.005 (0.001)	<0.001	1.005 (1.002-1.007)	0.001 (0.001)	0.583	1.001 (0.998-1.003)
**% missed appointments[l]**	-0.023 (0.004)	<0.001	1.024 (1.016-1.029)	-0.017 (0.003)	<0.001	1.017 (1.011-1.024)
**Mean number of treatment appointments**	0.025 (0.017)	0.157	1.025 (0.991-1.061)	0.036 (0.017)	0.031	1.037 (1.003-1.071)
**Mean number of days before entering treatment[l]**	-0.002 (0.001)	0.004	1.002 (1.001-1.003)	-0.002 (0.001)	<0.001	1.002 (1.002-1.004)
**Index of Multiple Deprivation[l]**	-0.007 (0.002)	<0.001	1.007 (1.003-1.011)	-0.010 (0.002)	<0.001	1.009 (1.006-1.013)
**Pre-treatment GAD score (anxiety cases)**	0.077 (0.048)	0.107	1.080 (0.983-1.185)	0.003 (0.046)	0.955	1.003 (0.917-1.096)
**Pre-treatment GAD score (depression cases)[l]**	-0.009 (0.020)	0.673	1.009 (0.969-1.049)	-0.003 (0.019)	0.872	1.003 (0.966-1.042)
**Pre-treatment PHQ score (anxiety cases)[l]**	<-0.001 (0.029)	0.990	1.000 (0.945-1.058)	-0.015 (0.027)	0.572	1.015 (0.962-1.072)
**Pre-treatment PHQ score (depression cases)[l]**	0.033 (0.027)	0.231	1.033 (0.979-1.091)	-0.009 (0.026)	0.735	1.009 (0.959-1.062)

S8. Multiple regressions including the effects of the pre-treatment symptom levels (PHQ and GAD) for depression and anxiety cases. ([l]) indicates where the reciprocal of the OR originally derived from the model is given.

PERMUTATION TESTS

We ran all analyses on a permuted dataset, which allowed us to verify that the significant association uncovered in our data arise due to a true relationship between the outcomes and the predictors, rather than excessive statistical power or other artefacts of the analytical methods. The outcome indicators (proportion of patients who reliably recover / improve) were sampled without replacement, and then randomly assigned to different CCGs. By doing so, we could break up the original associations between those outcomes and the predictors of our interest, and test whether our analyses would produce significant results in the absence of the original links between the outcomes and predictors. Significant results would suggest that the original results likely represent statistical artefact, rather than a true effect. On the other hand, obtaining non-significant results suggests that the significant associations uncovered in the non-permuted dataset reflect true relationships. Except for the initial permutation step, all analyses were as described in the main manuscript. For all sets of analyses, we produced ten dataset permutations, i.e. all analyses were re-run ten times, each with slightly different combinations of outcomes and predictors. P-values produced by each of those were then averaged, and are presented in the table below.

	Reliable Improvement		Reliable Recovery	
	2014/15	2015/16	2014/15	2015/16
**% patients with problem descriptor completeness**	0.47	0.41	0.51	0.56
**% patients entering treatment who receive a course of treatment**	0.50	0.62	0.25	0.59
**% missed appointments**	0.54	0.51	0.42	0.48
**Mean number of treatment appointments**	0.48	0.55	0.54	0.51
**Mean number of days before entering treatment**	0.73	0.51	0.59	0.68
**Index of Multiple Deprivation**	0.49	0.54	0.51	0.37

S9. Averaged p-values from the permutation tests for all beta regression (single-year) analyses.

COMPARISON BETWEEN BETA AND LOGISTIC REGRESSION

The four tables below (S10-S13) show a comparison of the results obtained using multiple beta and logistic regressions for the data for 2014/15 and 2015/16. While the effect sizes are very similar, logistic regression estimates tend to have narrower confidence intervals, and therefore are more significant than the results obtained using beta regression. This is due to different assumptions about the distribution underlying the data distribution (respectively, binomial and beta distribution). All the predictors that are significant in the beta regressions are also significant in the logistic regressions.

	Reliable Recovery 2014/15			
	Beta regression		Logistic regression	
	OR	p	OR	p
**% patients with problem descriptor completeness**	1.002 (1.000-1.003)	0.008	1.002 (1.001-1.002)	<0.001
**% patients entering treatment who receive a course of treatment**	1.004 (1.001–1.007)	0.016	1.002 (1.002-1.003)	<0.001
**% missed appointments[I]**	1.014 (1.005-1.022	0.003	1.010 (1.008-1.012)	<0.001
**Mean number of treatment appointments**	1.041 (1.003-1.080)	0.032	1.028 (1.022-1.036)	<0.001
**Mean number of days before entering treatment[I]**	1.002 (1.001-1.004)	0.015	1.001 (1.001-1.002)	<0.001
**Index of Multiple Deprivation[I]**	1.009 (1.005–1.013)	<0.001	1.011 (1.010-1.012)	<0.001

S10. Comparison of the results for predictors of Reliable Recovery in 2014/15 dataset obtained using multiple beta and logistic regressions. ([I]) indicates where the reciprocal of the OR originally derived from the model is given.

	Reliable Improvement 2014/15			
	Beta regression		Logistic regression	
	OR	p	OR	p
% patients with problem descriptor completeness	1.001 (0.999-1.002)	0.269	1.001 (1.000-1.001)	<0.001
% patients entering treatment who receive a course of treatment	1.009 (1.006-1.012)	<0.001	1.003 (1.003-1.004)	<0.001
% missed appointments[I]	1.015 (1.006-1.024)	<0.001	1.006 (1.005-1.007)	<0.001
Mean number of treatment appointments	1.027 (0.990-1.065)	0.162	1.014 (1.009-1.019)	<0.001
Mean number of days before entering treatment[I]	1.003 (1.002-1.005)	<0.001	1.001 (1.001-1.002)	<0.001
Index of Multiple Deprivation[I]	1.002 (0.998-1.006)	0.296	1.001 (1.001-1.002)	<0.001

S11. Comparison of the results for predictors of Reliable Improvement in 2014/15 dataset obtained using multiple beta and logistic regressions. ([I]) indicates where the reciprocal of the OR originally derived from the model is given.

	Reliable Recovery 2015/16			
	Beta regression		Logistic regression	
	OR	p	OR	p
% patients with problem descriptor completeness	1.001 (1.000-1.002)	0.083	1.001 (1.001-1.001)	<0.001
% patients entering treatment who receive a course of treatment	1.001 (0.999-1.002)	0.478	1.002 (1.001-1.002)	<0.001
% missed appointments[I]	1.017 (1.011-1.025)	<0.001	1.014 (1.013-1.016)	<0.001
Mean number of treatment appointments	1.017 (0.987-1.048)	0.276	1.022 (1.015-1.030)	<0.001
Mean number of days before entering treatment[I]	1.002 (1.001-1.004)	<0.001	1.002 (1.001-1.002)	<0.001
Index of Multiple Deprivation[I]	1.010 (1.007-1.013)	<0.001	1.008 (1.008-1.009)	<0.001

S12. Comparison of the results for predictors of Reliable Recovery in 2015/16 dataset obtained using multiple beta and logistic regressions. ([I]) indicates where the reciprocal of the OR originally derived from the model is given.

	Reliable Improvement 2015/16			
	Beta regression		Logistic regression	
	OR	p	OR	p
**% patients with problem descriptor completeness**	1.001 (1.000-1.002)	0.110	1.001 (1.001-1.001)	<0.001
**% patients entering treatment who receive a course of treatment**	1.006 (1.003-1.008)	<0.001	1.006 (1.006-1.007)	<0.001
**% missed appointments**	1.021 (1.014-1.027)	<0.001	1.017 (1.016-1.019)	<0.001
**Mean number of treatment appointments**	1.011 (0.978-1.046)	0.508	1.017 (1.010-1.024)	<0.001
**Mean number of days before entering treatment**	1.002 (1.001-1.004)	<0.001	1.002 (1.001-1.003)	<0.001
**Index of Multiple Deprivation**	1.004 (1.001-1.007)	0.018	1.002 (1.001-1.003)	<0.001

S13. Comparison of the results for predictors of Reliable Improvement in 2015/16 dataset obtained using multiple beta and logistic regressions.

WHAT HAPPENS TO THE WHOLE COHORT OF IAPT REFERRALS?

NHS Digital's IAPT Annual Report for 2015/16 includes detailed information on what happens to the whole cohort of IAPT referrals. The Table below, which is a simplified version of Table 4a in the report, shows the overall flow. Inspection of the discharge codes for referrals suggests that 81% of patients who were referred and assessed as potentially suitable for treatment in the services went on to have a course of treatment.

	**Number**	**% of referrals suitable for treatment**
Total referrals in 2015/16	1,299,525	
Never seen in service	405,974	
Seen once and appropriately not treated (treatment not necessary or unsuitable)	230,464	
Seen once and not treated but could have been as seemed suitable	125,956	19%
Treated in the service	537,131	81%
